# Sterol Biosynthesis Is Required for Heat Resistance but Not Extracellular Survival in *Leishmania*

**DOI:** 10.1371/journal.ppat.1004427

**Published:** 2014-10-23

**Authors:** Wei Xu, Fong-Fu Hsu, Eda Baykal, Juyang Huang, Kai Zhang

**Affiliations:** 1 Department of Biological Sciences, Texas Tech University, Lubbock, Texas, United States of America; 2 Department of Internal Medicine, Washington University School of Medicine, Saint Louis, Missouri, United States of America; 3 Department of Physics, Texas Tech University, Lubbock, Texas, United States of America; University of Dundee, United Kingdom

## Abstract

Sterol biosynthesis is a crucial pathway in eukaryotes leading to the production of cholesterol in animals and various C24-alkyl sterols (ergostane-based sterols) in fungi, plants, and trypanosomatid protozoa. Sterols are important membrane components and precursors for the synthesis of powerful bioactive molecules, including steroid hormones in mammals. Their functions in pathogenic protozoa are not well characterized, which limits the development of sterol synthesis inhibitors as drugs. Here we investigated the role of sterol C14α-demethylase (C14DM) in *Leishmania* parasites. C14DM is a cytochrome P450 enzyme and the primary target of azole drugs. In *Leishmania*, genetic or chemical inactivation of C14DM led to a complete loss of ergostane-based sterols and accumulation of 14-methylated sterols. Despite the drastic change in lipid composition, C14DM-null mutants (*c14dm* ^−^) were surprisingly viable and replicative in culture. They did exhibit remarkable defects including increased membrane fluidity, failure to maintain detergent resistant membrane fraction, and hypersensitivity to heat stress. These *c14dm* ^−^ mutants showed severely reduced virulence in mice but were highly resistant to itraconazole and amphotericin B, two drugs targeting sterol synthesis. Our findings suggest that the accumulation of toxic sterol intermediates in *c14dm* ^−^ causes strong membrane perturbation and significant vulnerability to stress. The new knowledge may help improve the efficacy of current drugs against pathogenic protozoa by exploiting the fitness loss associated with drug resistance.

## Author Summary

*Leishmania* parasites are transmitted through the bite of sandflies causing a spectrum of serious diseases in humans. Current drugs are inadequate and no safe vaccine is available. These parasites produce different types of sterols from humans, making the sterol synthesis pathway a valuable target of selective inhibitors. However, functions of sterols and sterol synthesis in protozoa are poorly understood, which hinders the development of new and improved treatments. In this study, we investigated the role of sterol C14α-demethylase, a key enzyme in sterol metabolism and the primary target of azole drugs. Loss of sterol C14α-demethylase completely altered the sterol composition in *Leishmania*, leading to increased membrane fluidity, failure to maintain lipid rafts, and hypersensitivity to heat stress. Despite these defects, null mutants of sterol C14α-demethylase were viable during the promastigote stage (found in sandflies) and could still cause disease in mice (although at a reduced capacity). Our findings provide direct evidence to support the role of specific sterols in membrane stability and stress response. The new knowledge may also help the development of new treatments or improve the efficacy of current drugs against pathogenic protozoa.

## Introduction

Leishmaniasis is a group of parasitic diseases infecting 10–12 million people in 88 countries [1]. It is caused by protozoan parasites of the genus *Leishmania* and transmitted through the bite of sandflies. During their life cycle, *Leishmania* parasites alternate between motile promastigotes which live in the midgut of sandflies and non-motile amastigotes which reside in the phagolysosome of mammalian macrophages. Depending on parasite species and host genetic factors, symptoms of leishmaniasis include localized skin sores, diffuse cutaneous lesions, severe mucosa destruction, and deadly visceral infections (kala azar) which damage the spleen, liver, and bone marrow [2]. Current treatments are often toxic, difficult to administer, and not cost-effective [3]. With drug resistance on the rise and no safe vaccine available, it is necessary to maintain a steady stream of new inhibitors and new biochemical targets to control these dangerous pathogens [4].

In eukaryotes, sterol biosynthesis is a vital pathway and an important source of antimicrobial targets. It consists of three stages: 1) the synthesis of isopentenyl pyrophosphate from acetyl CoA or an alternative carbon source such as leucine in trypanosomatids [5]; 2) the condensation of isopentenyl pyrophosphate and dimethylallyl pyrophosphate to form squalene; and 3) the cyclization of squalene into lanosterol, which is then converted into final products such as cholesterol, ergosterol, and phytosterol (Fig. S1) [6], [7]. Along with sphingolipids, sterols are tightly packed into ordered membrane microdomains or lipid rafts, which can be isolated as detergent resistant membrane fractions (DRMs) serving as scaffolds to support membrane integrity and signal transduction [8], [9]. In *Saccharomyces cerevisiae*, ergosterol synthesis is implicated in cell growth, ethanol resistance [10], heat shock response [11], and gene expression [12]. In mammals, cholesterol is a vital constituent of cell membrane and a key component of lipoprotein particles. It is also the precursor for the synthesis for various steroid hormones [13]. Precise functions of sterol synthesis in protozoa, however, are not well-characterized.

Similar to fungi, trypanosomatid pathogens including *Trypanosoma brucei*, *Trypanosoma cruzi*, and various *Leishmania* species synthesize C24-alkylated, ergostane-based sterols [14] (Fig. S1). Although the early steps of sterol synthesis (prior to zymosterol) are conserved in most eukaryotes, structural differences between mammalian enzymes and microbial enzymes can be exploited to produce selective drugs. Enzymes involved in the late steps of sterol pathway could also be valuable targets because mammalian cells do not synthesize ergostane-based sterols. Indeed, multiple classes of compounds targeting sterol biosynthesis exhibit good anti-trypanosomatid activities *in vitro* although their efficacies *in vivo* are often unsatisfactory. Examples include 3-(biphenyl-4-yl)-3-hydroxyquinuclidine which blocks the activity of squalene synthase (E.C. 2.5.1.21) [15], terbinafine which inhibits squalene epoxidase (EC 1.14.99.7) [16], [17], various azole drugs which target sterol 14-alpha-demethylase (C14DM, EC 1.14.13.70) [18]–[20], and azasterol which interferes the C24-alkylation of sterol precursor [21], [22]. Amphotericin B (Amp B) is another antifungal which binds to ergosterol or other ergostane-based sterols leading to pore formation on the plasma membrane [23], [24]. It possesses potent anti-*Leishmania* activity and is widely used as the drug of choice to treat antimony-resistant parasites [25]. Despite the promise, the underlying mechanism of how the alteration in sterol composition leads to growth retardation and/or parasite death is not well understood, which hinders the development of new and improved treatments [26]–[28].

The primary target of azole drugs is C14DM (known as CYP51 in animals and ERG11 in yeast), an evolutionarily conserved, heme-dependent, cytochrome P450 enzyme present in fungi, plants, mammals, and trypanosomatids [29] (Fig. S1). The reaction catalyzed by C14DM consists of three steps: the initial oxygenation of 14α-methyl group (–CH_3_) to 14α-alcohol (–CH_2_OH), further oxidation to 14α-aldehyde (–CHO), and finally the elimination of formic acid leading to the formation of C14-15 double bond in the sterol core [30]. Mouse C14DM is essential for embryogenesis, as deletion of this gene leads to embryonic lethality at day 15 [31]. In *S. cerevisiae*, null mutants of ERG11 require exogenous ergosterol to survive and only grow in the absence of oxygen or in the presence of a suppressor mutation in sterol C5-desaturase (ERG3, an enzyme upstream of C14DM) [12], [32]–[34]. While C14DM appears to be indispensable in mammals and fungi, azole drugs exhibit higher affinity for fungal enzymes over mammalian orthologs which contributes to their selectivity [35].

The C14DMs from several trypanosomatids have been cloned and biochemically characterized [36]–[38]. Significant efforts have been devoted to identify new and better C14DM inhibitors as anti-*T. cruzi* agents [18], [39]–[41]. Biochemical and structural studies of the C14DM from *Leishmania infantum* indicate that this enzyme prefers C4-monomethylated sterol substrates (such as 4, 14-dimethyl zymosterol), although it also metabolizes C4-dimethylated sterols (e.g. lanosterol) and C4-desmethylated sterols (e.g. 14α-methylzymosterol) with lower efficiency [38] (Fig. S1). This type of substrate preference is similar to the C14DMs in plants and *T. brucei* [37], [42] but distinct from that in *T. cruzi* which favors C4-dimethylated sterols [38], [43]. Meanwhile, the C14DMs in mammals and fungi provide rapid demethylation of sterol substrates without obvious restriction (regarding C4-methylation) [38].

The goal of our study is to address the following important yet still unanswered questions about sterol metabolism in *Leishmania*: Is C14DM essential for the promastigote stage (found in sandflies) and amastigote stage (found in mammals)? What is the role of sterol synthesis in the organization of plasma membrane? Is C14DM the primary target of azoles? How to improve the efficacy of current sterol synthesis inhibitors? To answer these questions, we generated and characterized a C14DM-null mutant in *Leishmania major*. Our results suggest that inactivation of C14DM severely disrupts the membrane stability of *Leishmania* parasites, probably due to the accumulation toxic sterol intermediates. Although this is not lethal by itself, it leads to extreme vulnerability to heat stress. The new knowledge will not only provide novel insight into the physiological role of sterol synthesis in *Leishmania* parasites, but also can guide the development of new treatments or to improve the efficacy of current antileishmanial drugs.

## Results

### Identification and targeted deletion of C14DM in *L. major*

*L. major* C14DM was identified from the TriTrypDB (gene ID: LmjF.11.1100) showing 28–33% identity to the C14DMs from human, fungi, and *Mycobacterium tuberculosis* (Fig. S2). Its syntenic orthologs are present in the genomes of *L. braziliensis*, *L. infantum*, *L. mexicana*, *T. brucei*, and *T. cruzi*. The *L. major* C14DM protein (479 aa) contains a potential N-terminal signal peptide (M1-F24) and motifs predicted to mediate sterol substrate binding (Y102–V113) and heme binding (G415–G424) [44] (Fig. S2).

**Figure 1:**
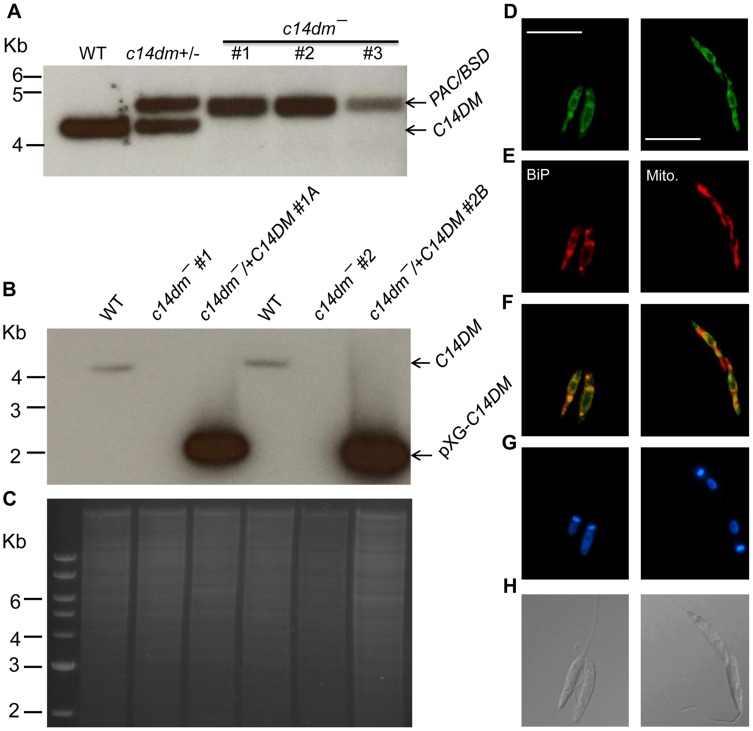
Targeted deletion and cellular localization of C14DM. (**A**–**C**) Genomic DNAs from *L. major* WT, *c14dm+/−* (heterozygous knockout), *c14dm* ^−^ (homozygous knockout) and *c14dm* ^−^ */+C14DM* (episomal add-back) parasites were subjected to Southern blot analyses, using radioactive probes from an upstream flanking region (**A**) or the open reading frame (**B**) of *C14DM*. (**C**) DNA loading control for **B** (ethidium bromide staining). (**D**–**H**) Immunofluorescence microscopy of *c14dm* ^−^/+*C14DM-GFP* promastigotes labeled with an ER marker (left panels) or Mitotracker (right panels). (**D**) GFP fluorescence (scale bars: 10 µm); (**E**) Anti-BiP staining (left panel; rabbit anti-*T. brucei* BiP antiserum followed by goat anti-rabbit IgG-Texas Red) or Mitotracker staining (right panel); (**F**) Merge of **D** and **E**; (**G**) Hoechst staining of DNA; (**H**) Differential interference contrast (DIC) images.

Using the targeted gene replacement approach [45], we were able to generate null mutants of C14DM (*c14dm* ^−^) in *L. major* promastigotes. Southern-blot confirmed the loss of endogenous *C14DM* alleles in three independent *c14dm* ^−^ clones (Fig. 1A–C). Add-back parasites (*c14dm* ^−^ */+C14DM*) were generated by introducing pXG-C14DM (a high copy number plasmid containing C14DM) into the mutants (Fig. 1B–C). To determine the localization of C14DM, a C14DM-GFP fusion protein was constructed and expressed in *c14dm* ^−^ promastigotes. The integrity and functionality of C14DM-GFP were confirmed by western blot and later by lipid analysis (Fig. S3). Immunofluorescence microscopy revealed a significant overlap between GFP fluorescence and the anti-BiP staining [46], but less so with the mitochondrial marker Mitotracker (Fig. 1D–H). These data suggest that C14DM is mainly located in the endoplasmic reticulum (ER) although a fraction of it may also reside in the mitochondrion. This localization is consistent with its predicted role in sterol biosynthesis and previous reports on C14DMs from *S. cerevisiae* and rat liver [47], [48].

### *C14dm* ^−^ promastigotes are viable and replicative but exhibit altered morphology and cytokinesis defect

**Figure 2:**
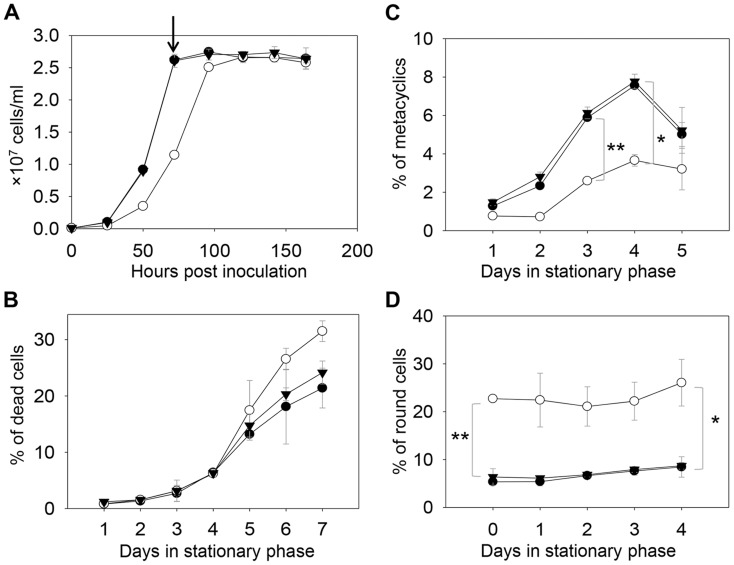
*C14dm* ^−^ mutants are viable but show defects in growth rate, cell shape and differentiation. (**A**) *L. major* promastigotes were inoculated at 1.0×10^5^ cells/ml and culture densities were measured every 24 hours. After entering stationary phase (72 hours post inoculation, marked by the arrow in **A**), percentages of dead cells (**B**), metacyclics (**C**) and round cells (**D**) were determined daily. Error bars represent standard deviations from 3 experiments (*: *p*<0.05, **: *p*<0.01). Black circle: WT; white circle: *c14dm* ^−^; black triangle: *c14dm* ^−^ */+C14DM*.

In *S. cerevisiae*, deletion of C14DM (*Δerg11*) led to ergosterol auxotrophy and cell death under aerobic conditions, possibly due to the production of oxygenated sterol intermediates [32]–[34]. Surprisingly, *L. major c14dm* ^−^ promastigotes were fully viable in culture during the replicative log phase although their doubling time (∼12 hours) was longer than that of wild type (WT) parasites (∼7 hours) (Fig. 2A–2B). Despite the slower growth rate, these mutants reached similar densities as WT parasites (2.3–3.0×10^7^ cells/ml) in stationary phase (Fig. 2A). In late stationary phase, *c14dm* ^−^ mutants had slightly more dead cells and produced less metacyclics (the non-replicative but highly infective forms [49]) than WT promastigotes (Fig. 2B–C). In addition, more round cells were detected in *c14dm* ^−^ mutants (20–30%) than in WT parasites (5–10%) in both log and stationary phase (Fig. 2D).

**Figure 3:**
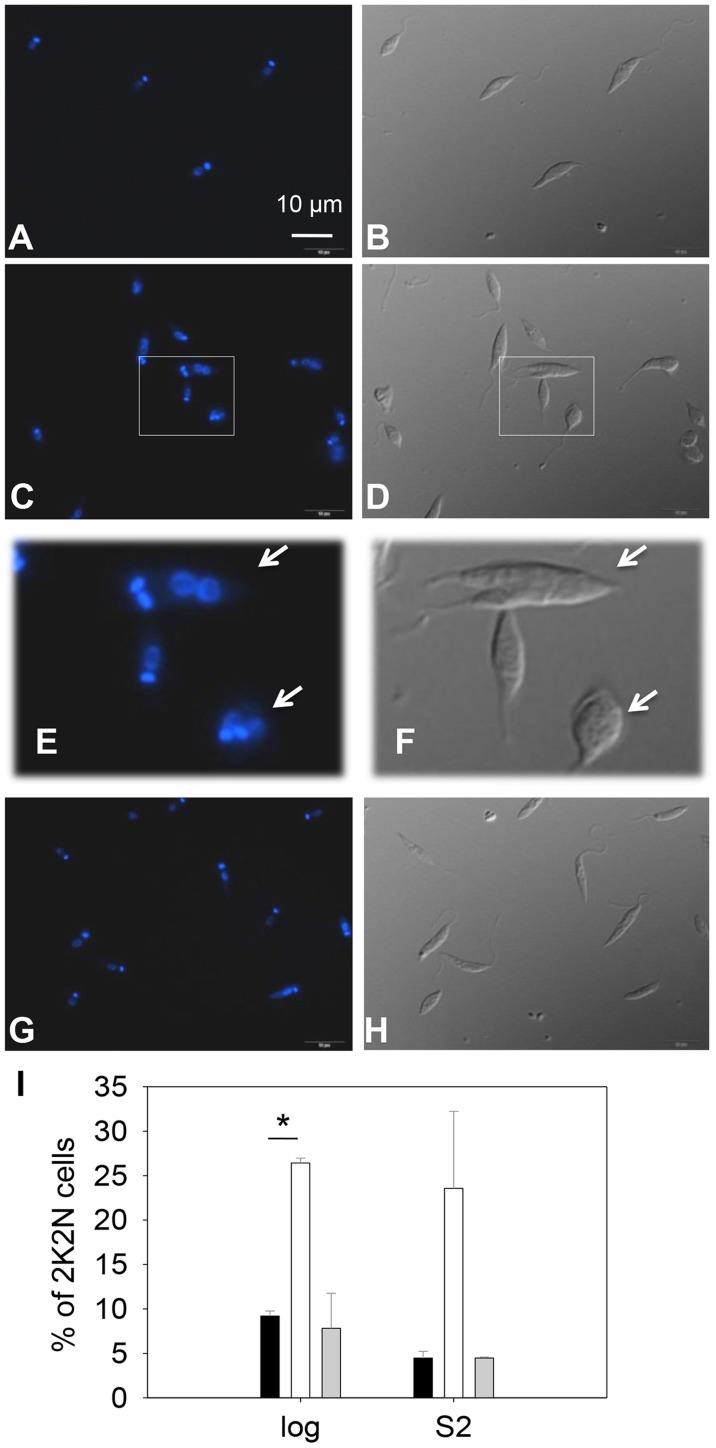
*C14dm* ^−^ promastigotes exhibit cytokinesis defects. Log phase promastigotes of WT (**A**–**B**), *c14dm* ^−^ (**C**–**F**), and *c14dm* ^−^ */+C14DM* (**G**–**H**) were stained with Hoechst 33242 and analyzed by fluorescence microscopy. **E** and **F** are enlargements of the boxed regions in **C** and **D**, respectively. DNA staining results were shown in **A**, **C**, **E** and **G**. DIC images were shown in **B**, **D**, **F**, and **H**. (**I**) Percentages of 2 kinetoplastids-2 nuclei (2K2N) cells were determined in log and day 2 stationary phase (S2) promastigote cultures. Black bars: WT, white bars: *c14dm* ^−^, grey bars: *c14dm* ^−^ */+C14DM* (*: *p*<0.05). Experiments were repeated three times (∼200 cells were counted for each cell type in every experiment) and error bars represent standard deviations. Examples of 2K2N cells are marked by arrows in **E** and **F**.

DNA staining revealed that 15–32% of *c14dm* ^−^ promastigotes had two kinetoplasts (containing mitochondrial DNA) and two nuclei (2K2N), whereas only 3–8% of WT parasites were 2K2N (Fig. 3). Similar results were observed in a cell cycle analysis of permeabilized promastigotes labeled with propidium iodide (Fig. S4). These data suggest that sterol synthesis is involved in maintaining normal cytokinesis in *Leishmania*. Defects manifested by *c14dm* ^−^ (growth delay, altered morphology, reduced metacyclogenesis, and overabundance of 2K2N cells) were completely reversed when *C14DM* expression was restored (*c14dm* ^−^ */+C14DM* in Figs. 2–3 and Fig. S4). Therefore, although C14DM is not required for promastigote survival or proliferation in culture, it is involved in the control of cell shape, differentiation, and division in *L. major*.

### *C14dm* ^−^ promastigotes have drastically altered sterol composition and show increased resistance to itraconazole (ITZ) and Amp B

**Figure 4:**
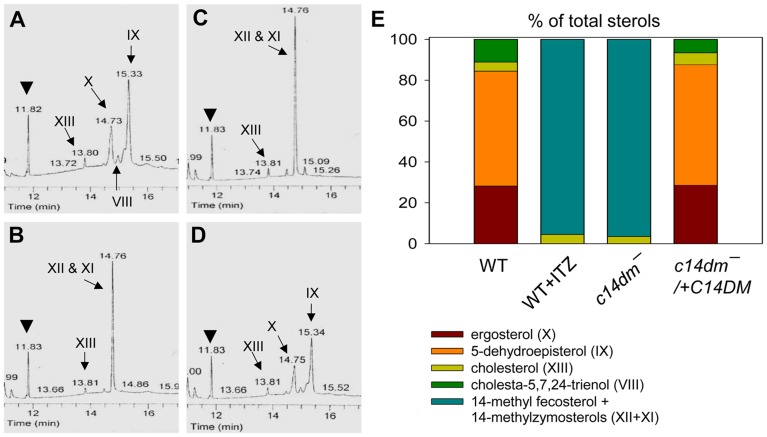
Accumulation of 14-methylated sterol intermediates and depletion of ergostane-based sterols in *c14dm* ^−^ mutants. (**A**–**D**) Partial GC chromatograms of lipids from WT (**A**), *c14dm* ^−^ (**B**), WT + 0.2 µM ITZ (**C**), and *c14dm* ^−^ */+C14DM* (**D**) promastigotes. Cholesta-3,5-diene (retention time = 11.82–11.83 minutes) was added to *Leishmania* samples prior to lipid extraction as an internal standard (arrowheads). Complete chromatograms were provided in Fig. S5–8. These analyses were repeated 3 times and percentages of major sterol species were quantified. Results from one representative experiment were summarized in **E**. The Roman numerals (VIII–XIII) represent sterol species (Fig. S1) and their corresponding peaks are indicated by arrows in **A**–**D**.

The effect of *C14DM* deletion on sterol synthesis was assessed by gas chromatography-mass spectrometry (GC-MS). Briefly, promastigote lipids were examined by total ion current (“A” in Fig. S5–Fig. S8), selected ion spectra (“B–F” in Fig. S5–Fig. S8), and the full mass spectra of major sterol species were acquired by electron ionization (Fig. S9). In WT parasites, four major sterol species were identified based on their retention time, formula weights, and electron impact mass spectra as the following: 5-dehydroepisterol (50–57%), ergosterol (22–28%), cholesta-5,7,24-trienol (6–10%), and cholesterol (3–5%) (Fig. 4A, 4E, Fig. S5 and Table S1). Deletion of *C14DM* led to a complete loss of ergostane-based sterols (5-dehydroepisterol, ergosterol, and episterol) and cholesta-5,7,24-trienol, but the level of cholesterol (salvaged from the medium) was not significantly affected (Fig. 4B, 4E, Fig. S6, and Table S1). Meanwhile, *c14dm* ^−^ mutants possessed a new, highly conspicuous sterol peak with a retention time of 14.76–14.78 on GC spectrum (Fig. 4B and Fig. S6). Selected ion analysis revealed that this peak was comprised of two lipid species with formula weights of 398.6 and 412.6 (Fig. S6C and 6F). Based on the role of C14DM in sterol synthesis, these lipids are predicted to be 14-methyl fecosterol (FW = 412.6, XII in Fig. S1) and 14-methyl zymosterol (FW = 398.6, XI in Fig. S1). Together these 14-methylated sterols constitute >95% of total sterols in *c14dm* ^−^ (Fig. 4B, 4E, and Table S1). Very similar results were observed when WT parasites of *L. major*, *L. donovani*, *L. mexicana*, and *L. amazonensis* were cultured in the presence of ITZ (3.3–200 nM), a C14DM inhibitor, for 2 days (Fig. 4C, 4E, Fig. S7, and Table S1). Parasites with episomal *C14DM* expression (*c14dm* ^−^ */+C14DM* and *c14dm* ^−^ */+C14DM-GFP*) had WT-like sterol composition, not elevated amounts of ergostane-based sterols (Fig. 4D, 4E, Fig. S8, Fig. S3B, and Table S1). This may reflect a limitation of substrates and/or a feedback regulation mechanism. It is also worth mentioning that deletion or overexpression of *C14DM* had no significant impact on the overall abundance of total sterols in *Leishmania* promastigotes (Table S1).

**Figure 5:**
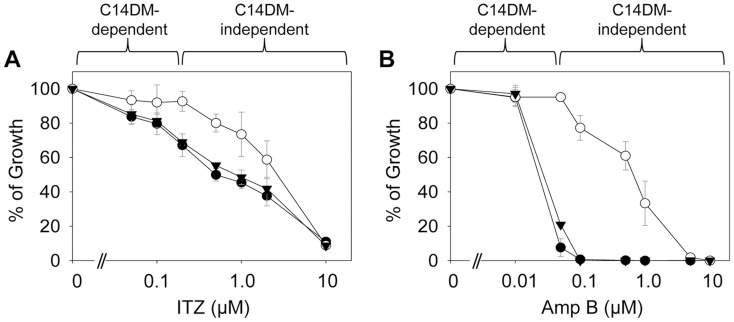
*C14dm* ^−^ mutants are more resistant to ergosterol synthesis inhibitors. Log phase promastigotes (black circle: WT; white circle: *c14dm* ^−^; black triangle: *c14dm* ^−^ */+C14DM*) were inoculated in M199 media (2×10^5^ cells/ml) in various concentrations of ITZ (**A**) or Amp B (**B**). Culture densities were determined after 48 hours and percentages of growth were calculated using cells grown in the absence of drugs as controls. Experiments were repeated three times and error bars represent standard deviations.

To test whether C14DM is the primary target of ITZ in *Leishmania*, promastigotes were inoculated in 0–10 µM of ITZ and culture densities were determined after 48 hours. For *L. major* WT and *c14dm* ^−^ */+C14DM* parasites, a dose-dependent response was observed (Fig. 5A); the IC25, IC50, and IC90 (concentrations required to inhibit growth by 25%, 50%, or 90%) were estimated to be 0.12 µM, 0.40 µM, and 10 µM, respectively (Fig. 5A and Table 1). Based on our sterol analysis, ITZ could shut down C14DM in WT *L. major* at fairly low concentrations (50 nM–0.2 µM) but only caused mild growth retardation (Fig. 4C, Table S1 and unpublished data). For *c14dm* ^−^ parasites, ITZ had negligible effect on growth at ≤0.2 µM, but did cause dose-dependent inhibition at >0.2 µM similar to WT parasites (Fig. 5A). For these mutants, the IC25, IC50, IC90 were around 0.60 µM, 2.0 µM, and 10 µM, respectively (Fig. 5A and Table 1). These data suggest that ITZ's mode of action is two-fold: at low concentrations (<0.2 µM), the drug mainly exerts its effect by blocking C14DM; and at high concentrations (>0.2 µM), it affects other targets beyond C14DM (Fig. 5A).

**Table 1:** Susceptibility of *c14dm* ^−^ mutants to ITZ and Amp B.

Drug	IC25 (µM ± SD)			IC50 (µM ± SD)			IC90 (µM ± SD)		
	WT	*c14dm* ^−^	Ratio	WT	*c14dm* ^−^	Ratio	WT	*c14dm* ^−^	Ratio
**ITZ**	0.12±0.10	0.60±0.20	1/5	0.40±0.10	2.0±0.3	1/5	10±1.4	10.0±1.0	1
**Amp B**	0.01±0.006	0.1±0.1	1/10	0.025±0.01	0.9±0.3	1/36	0.05±0.015	5.0±0.4	1/100

*L. major* WT or *c14dm* ^−^ promastigotes were inoculated in M199 medium at 2.0×10^5^ cells/ml in various concentrations of ITZ or Amp B. Culture densities were examined after 48 hours and IC values to achieve 25%, 50%, and 90% inhibition were determined by comparing to control cells grown in the absence of inhibitors. SD: standard deviations from 3 independent experiments. Ratios of IC (WT)/IC (*c14dm* ^−^) were indicated.

Amp B is another widely-used antifungal/antiprotozoal compound. It binds membrane sterol leading to the formation of channels and subsequent cell lysis. As shown in Fig. 5B and Table 1, *c14dm* ^−^ mutants were extremely resistant to Amp B as their IC values were 10–100 times higher than those of WT and *c14dm* ^−^/*+C14DM* parasites. These findings support the notion that Amp B targets ergostane-based sterols more efficiently than cholesterol-like sterols, which confers selectivity [50], [51]. Similar to ITZ, Amp B exhibited a biphasic inhibition on *L. major* growth: a C14DM-dependent phase at low concentrations (<0.1 µM) and a C14DM-independent phase at high concentrations (Fig. 5B). Together, these data indicate that: 1) loss of ergostane-based sterols (through genetic or chemical inactivation of C14DM) is not detrimental to promastigotes in culture; and 2) ITZ and Amp B have additional targets in *Leishmania* beyond the sterol synthesis pathway.

### *C14dm* ^−^ mutants exhibit significantly altered cell membrane organization

**Figure 6:**
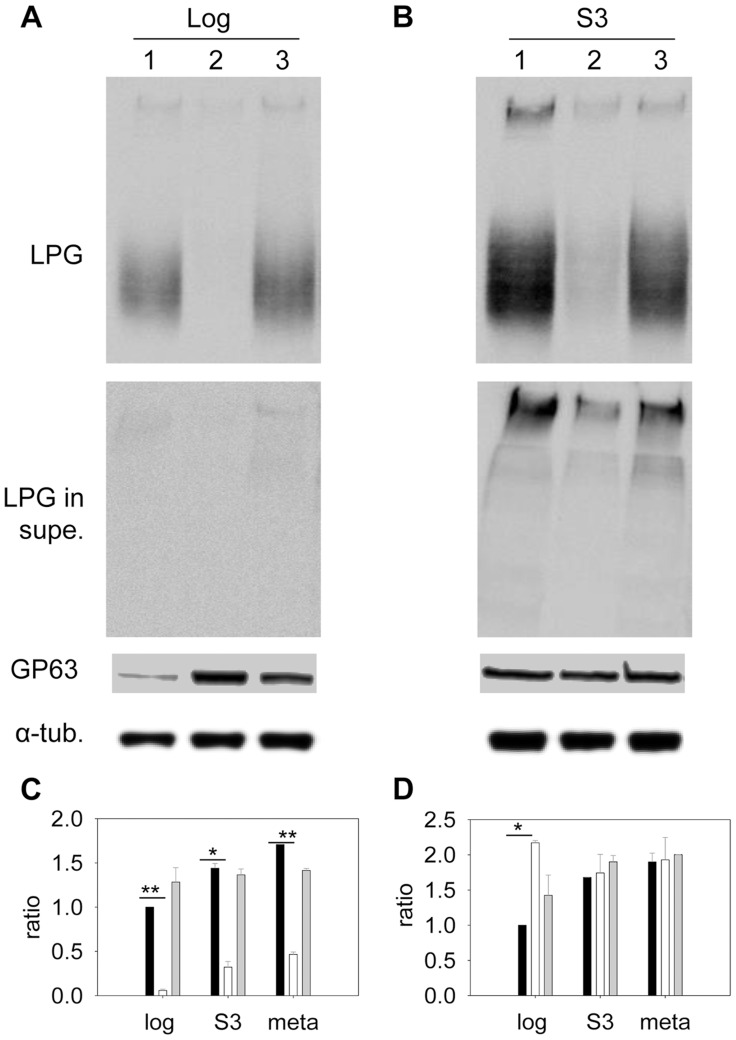
Altered expression of LPG and GP63 in *c14dm* ^−^ mutants. (**A**–**B**) Whole cell lysates or culture supernatants (LPG in supe.) from log phase (**A**) or day 3 stationary phase (**B**) promastigotes (lane 1: WT, lane 2: *c14dm* ^−^, lane 3: *c14dm* ^−^ */+C14DM*) were analyzed by Western blot, using antibodies against LPG, GP63, or α-tubulin. (**C**–**D**) The relative abundance of LPG (**C**) and GP63 (**D**) was normalized in log phase, stationary phase, and metacyclic parasites (black bars: WT; white bars: *c14dm* ^−^; grey bars: *c14dm* ^−^ */+C14DM*). Error bars represent standard deviations from 3 experiments (*: *p*<0.05, **: *p*<0.01).

Sterols are key stabilizers of biological membranes. Along with sphingolipids, they promote the formation of ordered membrane microdomains or lipid rafts [9], [52]. The unusual sterol profile in *c14dm* ^−^ prompted us to investigate whether sterol synthesis affects the expression and organization of membrane-bound, GPI-anchored virulence factors such as lipophosphoglycan (LPG) and GP63 (an abundant metalloprotease). In both log phase and stationary phase, *c14dm* ^−^ mutants had much less LPG than WT and *c14dm* ^−^ */+C14DM* cells based on western-blot (10–25%, Fig. 6A–C). This was not due to increased shedding/secretion, as the LPG in *c14dm* ^−^ culture supernatant was also low (Fig. 6A–B). Similar results were observed by immunofluorescence microscopy and flow cytometry using an anti-LPG antibody (Fig. S10). Meanwhile, these mutants contained more GP63 than WT (∼two-fold increase) in log phase but not in stationary phase or metacyclic promastigotes (similar to WT in these stages; Fig. 6A, B, D and Fig. S11). Therefore, changes in sterol composition do affect the steady state level of GPI-anchored virulence factors.

**Figure 7:**
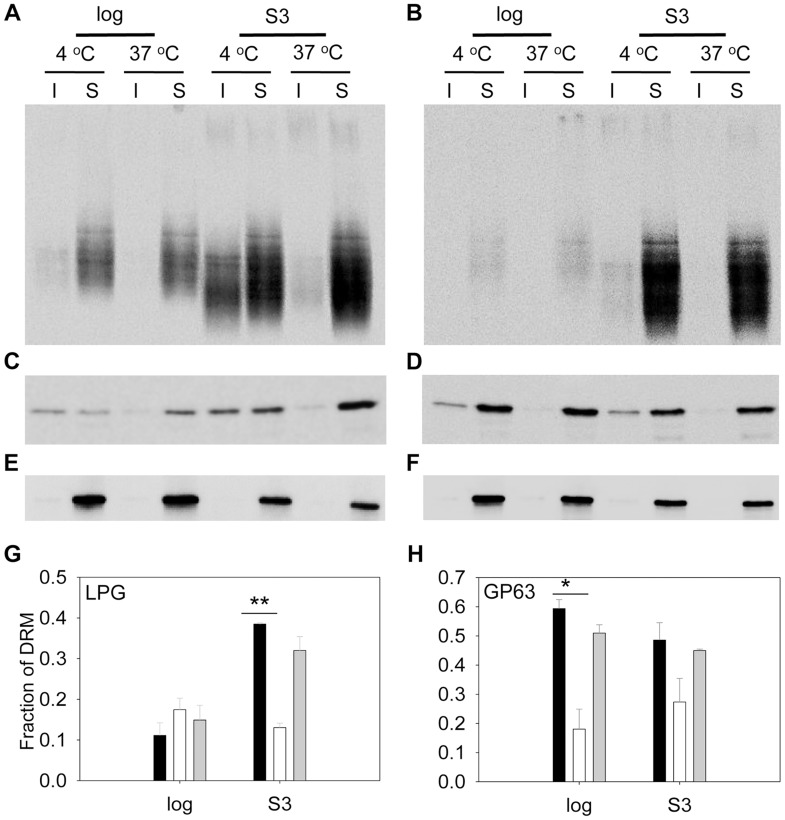
*C14dm* ^−^ mutants show reduced LPG and GP63 levels in the detergent resistant membrane (DRM) fractions. (**A**–**F**) Log phase and day 3 stationary phase cell lysates from WT (**A**, **C**, **E**) or *c14dm* ^−^ (**B**, **D**, **F**) promastigotes were extracted with 1% triton X100 at 4°C or 37°C. Both insoluble (I) and soluble (S) materials were analyzed by Western blot, using antibodies against LPG (**A**–**B**), GP63 (**C**–**D**) or HSP83 (**E**–**F**). Fractions of LPG and GP63 in DRM were quantified and summarized in **G** and **H**, respectively. Black bars: WT, white bars: *c14dm* ^−^, grey bars: *c14dm* ^−^ */+C14DM*. Error bars represent standard deviations from 3 experiments (*: *p*<0.05, **: *p*<0.01).

We also assessed the abundance of LPG and GP63 in liquid-ordered membrane microdomains by examining the DRMs. In mammalian cells and trypanosomatids, GPI-anchored macromolecules tend to be segregated in DRMs at 4°C (but not 37°C), which may reflect their association with cholesterol/sphingolipid- rich domains (lipid rafts) [9], [53]. In WT parasites, LPG was enriched in DRM in late stationary phase (35–38% of total LPG) but not in log phase (only 9–14% of total LPG) (Fig. 7A and G), indicative of a plasma membrane remodeling process during promastigote development as previously proposed [54]. Differing from LPG, GP63 had a clear association with DRM (50–60% of total GP63) in both log phase and stationary phase (Fig. 7C and H), suggesting that it is a constitutive component of lipid rafts. As a control, the cytosolic protein HSP83 was not found in DRM (Fig. 7E) [55]. Importantly, loss of C14DM reduced the DRM-association of GP63 in log phase and stationary phase (from 50–60% in WT to 20–32% in *c14dm* ^−^, Fig. 7C, D, and H); *c14dm* ^−^ mutants also had less LPG in DRM than WT during stationary phase (12–15% in *c14dm* ^−^ versus 35–38% in WT; Fig. 7A, B, and G); and restoration of C14DM expression reversed these defects (Fig. 7G and H). Collectively, these data indicate that ergostane-based sterols are critical not only for the synthesis and/or trafficking of GPI-anchored virulence factors, but also for their association with liquid-ordered microdomains.

### *C14dm* ^−^ mutants are highly attenuated in virulence

**Figure 8:**
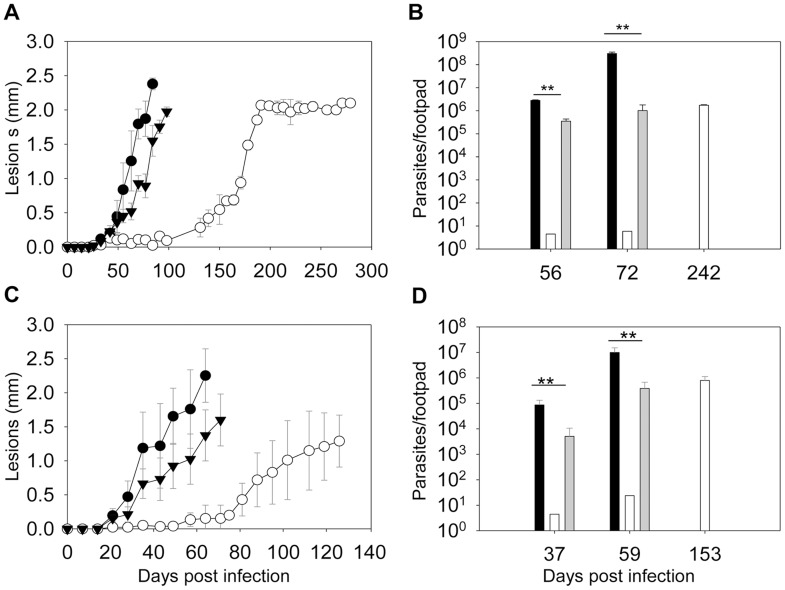
*C14dm* ^−^ mutants show severely attenuated virulence in BALB/c mice. BALB/c mice were infected with metacyclics (2×10^5^ parasites/mouse) (**A–B**) or lesion-derived amastigotes (2×10^4^ parasites/mouse) (**C–D**). Footpad lesions were recorded weekly and shown in **A** and **C** (black circle: WT; white circle: *c14dm* ^−^; black triangle: *c14dm* ^−^ */+C14DM*). Parasite numbers in the infected footpads were determined at the indicated times by limiting dilution assay and summarized in **B** and **D** (black bars: WT, white bars: *c14dm* ^−^, grey bars: *c14dm* ^−^ */+C14DM*). Error bars represent standard deviations from 5 mice in each group (**: *p*<0.01).

To determine whether ergosterol synthesis is required for *Leishmania* survival in mammals, metacyclics were isolated from stationary phase promastigotes and injected into the footpads of BALB/c mice. As indicated in Fig. 8A, WT and *c14dm* ^−^ */+C14DM* parasites caused rapid progression of lesions and all the mice had to be euthanized within 100 days post infection due to severe pathology. In contrast, mice infected by *c14dm* ^−^ did not show any disease for the first 120 days and it took them 180–200 days to develop large lesions (∼2.0 mm). The parasite loads in *c14dm* ^−^-infected mice were also significantly lower than those infected by WT or *c14dm* ^−^ */+C14DM* parasites at the same time (Fig. 8B). Similar results were obtained when BALB/c mice were infected with lesion-derived amastigotes of WT, *c14dm* ^−^, *c14dm* ^−^ */+C14DM* parasites (Fig. 8C–D). While LPG is an important virulence factor for *L. major* promastigotes, it is not required for the infectivity of amastigotes [56]. Thus, the reduced virulence of *c14dm* ^−^ cannot be solely attributed to LPG deficiency. Besides mouse infection, we also examined the ability of *c14dm* ^−^ mutants to parasitize primary murine macrophages *in vitro*. Comparing to WT and *c14dm* ^−^ */+C14DM* parasites, *c14dm* ^−^ mutants survived poorly in BALB/c macrophages (Fig. S12). Together, these findings demonstrate that C14DM is extremely important for *Leishmania* to effectively survive, proliferate, and cause disease in the mammalian host.

The fact that *c14dm* ^−^ mutants could still cause disease (at a reduced capacity nonetheless) suggests sterol synthesis is not absolutely essential for *Leishmania* during the mammalian stage. To investigate the effect of C14DM-deletion on the sterol composition of amastigotes, we isolated WT and *c14dm* ^−^ amastigotes from footpad lesions (Fig. S13) and examined their lipid contents by GC-MS (Fig. S14–15). For comparison, we also extracted lipids from uninfected mouse footpad tissue (Fig. S16) and promastigotes (Fig. S17–18; the RT values here were different from the ones in Figs. 4 and S5–S8 because a different GC column was used). Cholesta-3,5-diene was added as an internal standard to both amastigote and promastigote samples (1.0×10^9^ molecules/amastigote and 2.0×10^7^ molecules/promastigote; RT = 11.00 in Fig. S14–18). In WT amastigotes, a very high level of cholesterol was evident (RT = 12.87 in Fig. S14A–B), whereas the ergostane-based sterols (ergosterol, 5-dehydroepisterol, and episterol) were almost undetectable (Fig. S14C–D; similar to uninfected mouse tissue in Fig. S16). Clearly, some of the cholesterol was not directly associated with amastigotes (instead from mouse cells; Fig. S13 and Fig. S16). Nonetheless, the lack of endogenous sterols suggests that *de novo* sterol synthesis is significantly downregulated in amastigotes. This was in sharp contrast to WT promastigotes which had much more ergostane-based sterols than cholesterol (Fig. S17 and Fig. S5). *C14dm* ^−^ amastigotes also contained an overwhelming amount of cholesterol (Fig. S15A–B), much more abundant than the 14-methyl sterols (Fig. S15D–E) which were dominant in promastigotes (RT = 13.69 in Fig. S18). Since *Leishmania* parasites do not synthesize cholesterol [14], [57], these results suggest that amastigotes acquire the majority of their sterols from the host rather than *de novo* synthesis.

### *C14dm* ^−^ mutants show reduced cell membrane rigidity and are extremely sensitive to heat stress

**Figure 9:**
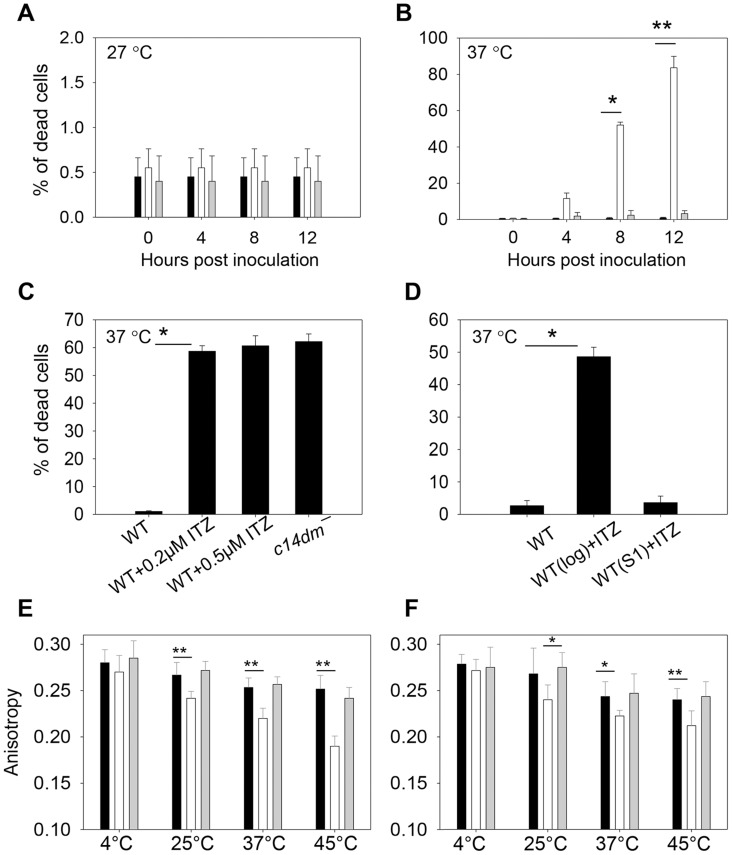
Inactivation of C14DM leads to extreme sensitivity to heat and increased membrane fluidity. (**A–B**) Promastigotes were cultured to stationary phase and half of the cells were maintained at 27°C (**A**) while the other half was incubated at 37°C/5% CO_2_ for 12 hours (**B**). Percentages of dead cells were determined every 4 hours (black bars: WT, white bars: *c14dm* ^−^, grey bars: *c14dm* ^−^ */+C14DM*). (**C**) WT (inoculated in the presence or absence of ITZ since early log phase at 2×10^5^ cells/ml) or *c14dm* ^−^ parasites were cultured to stationary phase and subjected to 37°C/5%CO_2_ treatment. Cell viability was measured after 8 hours. (**D**) ITZ (0.5 µM) was added to either early log phase (2×10^5^ cells/ml) or day 1 stationary phase (S1, ∼2.5×10^7^ cells/ml) WT cultures. After 2 days of incubation, cells were subjected to 37°C/5%CO_2_ treatment for 8 hours before the percentages of dead cells were measured. (**E–F**) Log phase (**E**) and stationary phase (**F**) promastigotes (black bars: WT, white bars: *c14dm* ^−^, grey bars: *c14dm* ^−^ */+C14DM*) were analyzed by anisotropy measurement. The rigidity of plasma membrane was determined by measuring the fluorescent depolarization of incorporated TMA-DPH at various temperatures. Experiments in **A–F** were repeated 3 times and error bars represent standard deviations (*: *p*<0.05, **: *p*<0.01).

Next we investigated whether sterol synthesis was involved in resistance to heat, acidic pH, and reactive oxygen intermediates/reactive nitrogen intermediates (ROIs/RNIs). In order to establish infection in mammals, *Leishmania* parasites must overcome these stress conditions. To examine if *C14DM* is required for heat tolerance, stationary phase parasites were incubated at either 27°C (the regular promastigote culture temperature) or 37°C (mimicking the mammalian body temperature). Most parasites were alive at 27°C as expected (Fig. 9A). At 37°C, however, 73–90% of *c14dm* ^−^ promastigotes were dead in 12 hours whereas the vast majority of WT and *c14dm* ^−^ */+C14DM* cells remained viable (Fig. 9B). Similar to *c14dm* ^−^, WT parasites grown in the presence of ITZ from log phase to stationary phase were hypersensitive to 37°C condition (Fig. 9C). In contrast, if WT parasites were cultured without ITZ to stationary phase and then treated with ITZ (which would not significantly affect sterol synthesis since most stationary phase cells were non-replicative), they did not show such defects (Fig. 9D). Therefore, it is the alteration of sterol composition (rather than other effects from ITZ) that is responsible for this hypersensitivity to high temperature.

To determine if the function of C14DM on heat resistance is conserved in other *Leishmania* species, we grew *L. mexicana*, *L. amazonensis* and *L. donovani* parasites in sub-lethal concentrations of ITZ (3.3 nM for *L. mexicana*, 25 nM for *L. amazonensis*, and 81 nM for *L. donovani*) which were sufficient to shut down ergostane-based sterol synthesis but only inhibit growth by ∼25% (Fig. S19A, Table S1 and S2). Similar to *c14dm* ^−^, these ITZ-treated parasites were extremely vulnerable to heat (Fig. S19B). Therefore, C14DM likely plays similar roles in multiple *Leishmania* species.

We also tested the ability of *c14dm* ^−^ mutants to withstand oxidative, nitrosative and acidic pH stress as previously described [58]. As shown in Fig. S20A–B, these mutants were slightly more sensitive to SNAP (a nitric oxide releaser) than WT and *c14dm* ^−^ */+C14DM* parasites (although the difference was not statistically significant), which might be due to their low LPG abundance (Fig. 6) [56]. Meanwhile, their resistance to H_2_O_2_ and acidic pH were normal (Fig. S20C–F).

Since sterols could function as stabilizers in lipid bilayer [59], we examined whether alteration in sterol composition affects the cell membrane fluidity of *c14dm* ^−^ mutants, which may be linked to their heat sensitivity and defects in forming DRM/rafts. To do so, promastigotes were labeled with TMA-DPH (a cationic lipophilic probe that diffuses into the outer leaflet of lipid bilayer) for 20 min at 4°C, 25°C, 37°C, or 45°C (>95% of cells were alive by propidium iodide staining). Plasma membrane fluidity was then determined by measuring the fluorescence depolarization of TMA-DPH as previously described [60]. As indicated in Fig. 9E–F, WT and *c14dm* ^−^ */+C14DM* parasites maintained their membrane fluidity at a reasonably stable level when the temperature rose from 4°C to 45°C (a high anisotropy value means the membrane is more rigid or less fluid). In contrast, the plasma membrane of *c14dm* ^−^ mutants became much more fluid at elevated temperatures (Fig. 9E–F). Therefore, defects in sterol synthesis may compromise cell membrane stability and rigidity at high temperatures, resulting in hypersensitivity to heat.

## Discussion

In this study, we investigated the role of C14DM in *L. major*, a vector-borne protozoan parasite responsible for cutaneous leishmaniasis. C14DM catalyzes the heme-dependent oxidative removal of 14α-methyl group from sterol intermediates, a key step in sterol biosynthesis. Deletion of C14DM in *L. major* results in a complete loss of ergostane-based sterols and significant accumulation of 14-methylated sterol intermediates. This drastic change of sterol composition leads to increased plasma membrane fluidity, failure to form normal DRM/lipid rafts, and extreme vulnerability to heat. Nonetheless, *c14dm* ^−^ mutants are fully viable and replicative as promastigotes in culture with only minor imperfections in growth rate, morphology and cytokinesis. They do exhibit marked defects in the synthesis and/or trafficking of GPI-anchored virulence factors and are more resistant to antifungals such as ITZ and Amp B. The infectivity of *c14dm* ^−^ mutants is greatly reduced but not completely abolished, suggesting that inhibition of C14DM by itself is not sufficient to eliminate *L. major* infection.

It is rather surprising that *Leishmania* promastigotes remain viable and proliferative without C14DM. In the absence of endogenous sterols, *c14dm* ^−^ mutants mainly accumulate 14-methylated intermediates. Similar results were observed when parasites were exposed to sub-lethal concentrations of azoles (Table S1) [57], [61]. Since the overall level of sterols is similar between WT and *c14dm* ^−^ parasites (Table S1; only the composition is altered), it appears that 14-methylfecosterol and 14-methylzymosterol could partially compensate the loss of ergostane-based sterol. Other membrane lipids such as sphingolipids, glycerophospholipids, and cholesterol (salvaged from the environment) may also help stabilize the plasma membrane in *Leishmania*. However, the aberrant sterol composition in *c14dm* ^−^ does have serious consequences as these mutants fail to maintain proper membrane rigidity at elevated temperatures, which probably contributes to their hypersensitivity to mild heat (although other mechanisms may also be involved). Inactivation of C14DM also seems to interfere with the formation of liquid-ordered microdomains, as the DRMs from *c14dm* ^−^ is depleted of GP63 and LPG which should be enriched in lipid rafts. One possibility is that protrusion of axial 14α-methyl group from the planar 4-ring core structure decreases the interaction between sterols and phospholipid side chains [62], [63]. Consequently, compared to regular sterols (which possess a smooth α-side), 14α-methylated sterols may be less efficient at promoting the condensation of lipid bilayer, leading to increased membrane fluidity (especially at elevated temperatures) in *c14dm* ^−^ [61], [64] [65], [66]. Additionally, the loss of ergostane-based sterols (besides the accumulation of 14α-methylated sterols) may also contribute to these membrane defects. It has been reported that ergosterol is more effective at promoting the liquid-ordered phase than lanosterol (which also contains the 14α-methyl group) [67], [68].

The altered shape of *c14dm* ^−^ mutants is likely caused by increased membrane permeability due to high fluidity, allowing more water penetration as previously shown in *S. cerevisiae* treated with fluconazole [69]. *C14dm* ^−^ mutants also have more 2K2N cells which have completed DNA replication but are slow to finish division, consistent with their prolonged doubling time. In mammalian cells, cholesterol starvation induced growth arrest at G2 phase and polyploidy formation [70], [71]. In *S. cerevisiae*, sterol depletion led to growth arrest at G1 stage [12]. Addition of cholesterol and ergosterol at hormonal amounts reversed these effects in mammalian cells and yeasts, respectively [12]. This indicates that in addition to its membrane function, sterols also possess a signaling role in fungi and mammals.

As promastigotes, *c14dm* ^−^ mutants cannot be rescued by exogenous ergosterol when provided at nM-µM range, suggesting that: 1) the accumulation of 14-methylated sterol intermediates (rather than the lack of ergostane-based sterols) is primarily responsible for the defects in membrane stability, heat resistance and replication; or 2) the uptake of ergosterol by *Leishmania* is insufficient although cholesterol can be incorporated into the membrane. Loss of C14DM also affects the synthesis and/or trafficking of major GPI-anchored virulence factors, as *c14dm* ^−^ mutants contain less LPG but more GP63 (only in the log phase) than WT parasites. Previous studies suggest that the synthesis of LPG and GP63 starts with a common pool of alkyl-acyl-PIs with long alkyl chains (C24:0/C26:0), followed by differential glycosylation and fatty acid remodeling in separate compartments [72], [73]. Alteration in sterol composition may compromise the vesicular trafficking or the proper compartmentalization of these pathways, causing abnormality in GPI-molecule synthesis.

The hypersensitivity of *c14dm* ^−^ mutants to heat is probably a key contributing factor to their severely reduced virulence in mice. The LPG deficiency could partially explain the virulence defect of *c14dm* ^−^ promastigotes but is unlikely to be a major factor for amastigotes since LPG is not required during the mammalian stage of *L. major* [74]. Besides heat tolerance, the synthesis of ergostane-based sterols is likely needed for other purposes. In *L. amazonensis*, ketoconazole (another azole drug targeting C14DM) treatment induced the appearance of large multivesicular bodies, increased amounts of lipid droplets and acidocalcisomes (calcium- and phosphate-rich organelles) [75], and alterations in the distribution and appearance of mitochondrial cristae [76], [77]. *L. amazonensis* parasites exposed to 22,26-azasterol, a sterol methyltransferase inhibitor, also exhibited profound morphological changes including mitochondrial swelling, increased number of acidocalcisomes, and the appearance of large, membranous bodies reminiscent of autophagic vesicles [22]. Comparing to *Leishmania* promastigotes, intracellular amastigotes show a global decrease in the uptake and utilization of glucose and amino acids, but are more dependent on mitochondrial metabolism (for TCA cycle and glutamine synthesis) [78]. Thus, perturbation of mitochondrial structure/function may be an underlying mechanism for the anti-proliferative effect of sterol synthesis inhibitors.

Importantly, after a delay of 70–120 days, *c14dm* ^−^ -infected mice started to show symptom (footpad swelling) and eventually produced lesions similar to WT-infected mice. Mutant parasites were also capable of proliferation after the initial delay. Promastigotes and amastigotes isolated from *c14dm* ^−^ -infected mice were still attenuated (Fig. 8), suggesting that this is not due to reversion or compensatory mutations. Hence, despite their profound defects, *c14dm* ^−^ mutants are still somewhat virulent. One possibility is that *Leishmania* amastigotes salvage huge amounts of host lipids including cholesterol and sphingolipids [79]–[81], which may alleviate the loss of *de novo* synthesis and/or accumulation of toxic sterols. This is supported by our amastigote lipid analysis which showed significant accumulation of cholesterol (host-derived) and only trace amount of endogenous sterols (Fig. S13–18).

The fact that *c14dm* ^−^ mutants are viable as promastigotes and infective in mice (at a reduced capacity) suggests that inhibition of C14DM by itself may not be sufficient to cure *Leishmania* infection. *In vitro*, azole drugs such as ketoconazole, fluconazole, itraconazole, and posaconazole have shown activity against the growth of *Leishmania* and *Trypanosoma cruzi* (responsible for Chagas disease), yet their *in vivo* efficacies remain somewhat unsatisfactory [39], [82]–[85]. These drugs are often limited by poor pharmacokinetics (difficulties in formulation, delivery and bioavailability) [86] and emergence of resistance (e.g. increased drug efflux and mutations in the target gene) [86]. Findings from our study suggest that the efficacy of azoles may improve if they are used in combination with localized heat treatment. Thus, although C14DM inhibition only exerts modest anti-*Leishmania* effect, it does make parasites vulnerable to other physical or chemical perturbations.

For *Leishmania* promastigotes, ITZ (and possibly other azoles) treatment seems to be mimic the effect of C14DM deletion at low concentrations but it clearly inhibits other unknown targets at high concentrations (Fig. 5A and Fig. S19A). Based on our findings, it may be worthwhile to explore whether inhibitors of sphingolipid/phospholipid synthesis can exacerbate the membrane instability of *c14dm* ^−^ mutants. If so, combined inhibition of multiple lipid synthesis pathways may have synergistic effect on parasite survival. Our findings also indicate that mutations in C14DM can confer significant resistance to Amp B, although the fitness costs associated with such mutations could be therapeutically exploited.

In summary, genetic or chemical inactivation of C14DM in *Leishmania* results in dramatic change in sterol composition, leading to DRM/raft disruption, increased membrane fluidity, and impairment in the synthesis and/or trafficking of GPI-anchored molecules. Ablating C14DM is not detrimental in *L. major*, perhaps due to the compensatory effect of other lipids, but does render parasites extremely vulnerable to heat. These findings may guide the development of new therapies which would improve the efficacies of current treatments and exploit the fitness cost of drug resistant strains. In addition, future studies will determine the mechanistic basis of *c14dm* ^−^ -associated defects, e.g. whether they are mainly caused by membrane perturbations or dysregulation of intracellular pathways. The viability of *c14dm* ^−^ mutants also provides a valuable platform to study the roles of DRM/rafts in crucial events such as vesicular trafficking and signaling. Finally, the interaction between *Leishmania* amastigotes and host cells at sterol metabolism, e.g. *de novo* synthesis vs salvage is another important topic worthy of further studies.

## Materials and Methods

### Materials

BALB/c (female, 7–8 weeks old) mice were purchased from Charles River Laboratories International (Wilmington, MA). All procedures involving mice were approved by the Animal Care and Use Committee at Texas Tech University (PHS Approved Animal Welfare Assurance No. A3629-01). Mice were housed and cared for in the facility operated by the Animal Care and Resources Center at Texas Tech University adhering to the *Guide for the Care and Use of Laboratory Animals* (the 8th Edition, NRC 2011) for animal husbandry. Reasonable efforts were made to minimize animal suffering. Anesthesia was applied through intra-peritoneal injection of ketamine hydrochloride (100 mg/kg)/xylazine (10 mg/kg). Euthanasia was achieved by asphyxiation through controlled flow of pure CO_2_.

Zymosterol, lanosterol, cholesterol, ergosterol and 5-dehydroergosterol were purchased from Avanti Polar Lipids (Birmingham, AL) as standards (to determine retention times) in gas chromatography-mass spectrometry (GC-MS) studies. Cholesta-3,5-diene was purchased from Sigma-Aldrich (St. Louis, MO) as an internal standard for quantitation in total ion current chromatograms. Itraconazole (ITZ) was purchased from LKT Laboratories, Inc. (St. Paul, MN). Amphotericin B (Amp B) and 30% H_2_O_2_ were purchased from EMD Chemicals, Inc. (San Diego, CA). 1-(4-Trimethylammoniumphenyl)-6-Phenyl-1,3,5-Hexatriene p-Toluenesulfonate (TMA-DPH) was purchased from Life Technologies Corporation (Grand Island, NY). All other chemicals were purchased from VWR International or Fisher Scientifics unless specified otherwise.

### Molecular constructs

The predicted open reading frame (ORF) of *L. major C14DM* (LmjF.11.1100) was amplified by PCR from *L. major* genomic DNA using primers #170/#171. The resulting 1.44 Kb DNA fragment was digested with *BglII* and cloned in the pXG vector [87] as pXG-*C14DM* (B294). A modified *C14DM-*ORF was amplified with primers #170/#333 to remove the stop codon and cloned into the pXG-*'GFP+* vector [87] to generate pXG-*C14DM-GFP* (B321), which was used to generate a C-terminal GFP fusion protein for the localization study.

The upstream and downstream flanking sequences (∼1 Kb each) of *C14DM* ORF were amplified with primers #172/#173 and primer #174/#175, respectively. These two PCR products were cloned together into pUC18. Genes conferring resistance to the puromycin (*PAC*) and blasticidin (*BSD*) were inserted between the upstream and downstream flanking sequences to generate pUC-KO-*C14DM::PAC* (B292) and pUC-KO-*C14DM::BSD* (B293). Primers used in this study were summarized in Table S3. All DNA constructs were confirmed by restriction enzyme digestion and sequencing.

### *Leishmania* culture, genetic manipulations, and Southern blot

*L. major* LV39 clone 5 (Rho/SU/59/P), *L.(L) amazonensis* (MHOM/BR/77/LTB0016), *L. (L) mexicana* M379 (MNYC/BZ/62/M379) and *L. donovani* 1S2D (MHOM/SD/62/1S) promastigotes were cultured at 27°C in M199 medium (pH 7.4) with 10% fetal bovine serum and additional supplements [88]. In general, log phase promastigotes refer to replicative parasites at densities lower than 1.0×10^7^ cells/ml, and stationary phase promastigotes refer to non-replicative parasites at densities higher than 2.0×10^7^ cells/ml. The infective metacyclic parasites (metacyclics) were isolated from stationary phase promastigotes using the density centrifugation method [89].

To generate *C14DM*-null mutants (*c14dm* ^−^ or *▵C14DM::PAC/▵C14DM::BSD*), the *C14DM* alleles from wild type *L. major* parasites (WT) were sequentially replaced by *PAC* and *BSD* resistance genes using the homologous recombination-based approach as previously described [90]. To confirm the loss of *C14DM*, genomic DNAs were digested with *SacI*, resolved on a 0.7% agarose gel, transferred to a nitrocellulose membrane, and hybridized with a [^32^P]-labeled DNA probe recognizing either the *C14DM* ORF or a ∼500-bp upstream region of *C14DM*. Blots were then visualized by radiography. The *c14dm* ^−^ mutants were maintained in media containing 10 µg/ml of puromycin and 10 µg/ml of blasticidin. To restore *C14DM* expression, pXG-*C14DM* or pXG-*C14DM-GFP* was introduced into *c14dm* ^−^ by electroporation and stable transfectants were referred to as *c14dm* ^−^ */+C14DM* or *c14dm* ^−^ */+C14DM-GFP*, respectively. Three independent *c14dm* ^−^ mutant clones were generated and their phenotypes were nearly identical. Therefore, *c14dm* ^−^ #1 and its add-back control were described in this study.

### Cell growth, stress response and drug sensitivity

To measure promastigote growth, parasites were inoculated in complete M199 medium at 1.0×10^5^ cells/ml. Culture density was determined at designated times using a hemacytometer. Percentages of round cells (defined as those with the long axis shorter than twice the length of the short axis) and dead cells were determined by microscopy and flow cytometry, respectively, as previously described [91].

To assess thermal tolerance, stationary phase promastigotes were incubated at either 27°C (the regular temperature) or 37°C/5%CO_2_ and cell viability were determined after 0–12 hours [58]. To measure sensitivity to oxidative and nitrosative stress, stationary phase promastigotes were incubated in various concentrations of H_2_O_2_ or *S*-nitroso-*N*-acetylpenicillamine (SNAP) [92]; cell density and viability were determined after 48 hours. To determine sensitivity to acidic pH, stationary phase promastigotes were inoculated in a pH 5.0 medium at 2.5×10^7^ cells/ml and culture densities were determined after 48 hours [58].

To test drug sensitivity, promastigotes were inoculated in M199 medium at 2.0×10^5^ cells/ml in the presence of ITZ (0–10 µM) or Amp B (0–10 µM). Culture densities were determined after 48 hours.

### Western blot, immunofluorescence microscopy and flow cytometry

To collect whole cell lysates, promastigotes were washed once in PBS and resuspended at 5.0×10^7^ cells/ml in 1× SDS sample buffer. Supernatants were collected from log and stationary phase cultures after centrifugation. To generate detergent resistant membrane fractions (DRMs), promastigotes were washed once in PBS and extracted with 1% of TritonX-100 (at 1.0×10^8^ cells/ml) for 10 minutes at 4°C or 37°C. Detergent-soluble and -insoluble fractions were separated by centrifugation at 14,000 g for 2 minutes. An equal volume of 2 × SDS sample buffer was added to the detergent soluble fraction and two volumes of 1 × SDS sample buffer were added to the detergent insoluble fraction [53]. Samples were boiled for 5 minutes before SDS-PAGE. After transfer to PVDF membranes, blots were probed with either mouse-anti-LPG monoclonal antibody WIC 79.3 (1∶1000) [93], or mouse-anti-GP63 monoclonal antibody #235 (1∶1000) [94], followed by a goat anti-mouse IgG conjugated with HRP (1∶2000). For C14DM-GFP, blots were probed with a rabbit anti-GFP HRP-conjugated antibody (1∶5000). For loading controls, blots were probed with a mouse-anti-α-tubulin antibody or a rabbit anti-*Leishmania* HSP83 antibody. A FluorChem E system (Protein Simple) was used to detect and quantify signals.

For LPG/GP63 localization, formaldehyde-fixed parasites were attached to poly-lysine coated cover slips and permeabilized with ice-cold ethanol. Cells were labeled with either mouse-anti-LPG antibody WIC79.3 or mouse-anti-GP63 antibody (both at 1∶2000 dilution in 2% bovine serum albumin prepared in PBS) for 20 minutes, and then incubated with a goat anti-mouse IgG-FITC (1∶1000 dilution) for 20 minutes. For C14DM-GFP localization, *c14dm* ^−^ */+C14DM-GFP* parasites were labeled with a rabbit anti-*T. brucei* BiP antiserum (1∶10,000) [46] for 30 minutes and then incubated with a goat anti-rabbit IgG-Texas Red antibody (1∶1000 dilution) for 30 minutes. For mitochondrial staining, 1×10^6^ parasites were centrifuged at 1000 g for 10 minutes, resuspended in 350 nM of Mitotracker Red 580 (Life technologies) in darkness; after 30 minutes, cells were washed in PBS once, and fixed with 3.7% formaldehyde; cells were then transferred to poly-L-lysine coated coverslips by centrifugation (462 g for 5 minutes), washed by 50% methanol, and stained with 1.0 µg/ml of Hoechst 33342 for 10 minutes. Images were acquired using an Olympus BX51 Upright Fluorescence Microscope equipped with a digital camera.

Flow cytometry analyses for cell viability, DNA content, and surface LPG expression were performed as previously described [53] [95], [96], using a BD Accuri C6 flow cytometer.

### Sterol analysis by gas chromatography/mass spectrophotometry (GC-MS)

Total lipids were extracted according to a modified Folch's protocol [97]. Briefly, promastigotes were resuspended in chloroform: methanol (2∶1) at 1.0×10^8^ cells/ml and vortexed for 30 seconds. An internal standard, cholesta-3,5-diene (FW = 368.84), was added to cell extract at 2.0×10^7^ molecules/cell (or 1.2 µg/10^8^ promastigotes). Cell debris was removed by centrifugation (1000 g for 10 minutes) and the supernatant was washed with 0.2 volume of 0.9% NaCl. After centrifugation, the aqueous layer was removed and the organic phase was dried under a stream of air. Lipid samples were then dissolved in methanol at the equivalence of 1.0×10^9^ cells/ml. Lesion amastigotes were purified from infected mice as previously described [98]. Amastigote lipids were then extracted following the same procedure as promastigote samples except that the internal standard (cholesta-3,5-diene) was provided at 1.0×10^9^ molecules/amastigote (due to the high cholesterol level) or 30 µg/5×10^7^ amastigotes/footpad. Lipid from uninfected mouse footpads also contained the internal standard (30 µg/footpad).

Electron impact GC/MS analyses of sterol lipids were performed on a Thermo Scientific ISQ (San Jose, CA) single-stage quadrupole mass spectrometer with Trace GC controlled by Thermo Xcalibur 2.1 software. The extract (1 µL) was injected in a splitless mode and analyzed by GC on a Phenomenex (Torrance, CA) ZB-50 column (15 m, 0.32 mm id, 0.5 µm film thickness). The initial temperature of GC was set at 100°C for 2 min, increased to 200°C at a rate of 50°C/min, and then raised to a final temperature of 300°C at a rate of 10°C/min (and then maintained at 300°C for 10 min). Temperatures of the injector, transfer line of the GC column, and of the ion-source were set at 280°C, 280°C, and 220°C, respectively. The full scan mass spectra (50 to 500 Dalton) or total ion current chromatograms were acquired at a rate of 1 scan/0.2 sec. Electron ionization mass spectra of major *Leishmania* sterols were performed at 70 eV. Pure sterol standards (zymosterol, lanosterol, cholesterol, ergosterol and 5-dehydroergosterol) were also analyzed to obtain their electron impact mass spectra and GC retention times.

### Macrophage infection and mouse footpad infection

Bone marrow derived macrophages were isolated from BALB/c mice as previously described [58]. Macrophage infection was performed using metacyclic promastigotes (opsonized with C57BL6 mouse serum) at a ratio of five parasites per macrophage [99].

Footpad infections of BALB/c mice were performed as previously described [58] using metacyclic promastigotes (2.0×10^5^ cells/mouse) or lesion-derived amastigotes (2.0×10^4^ cells/mouse) [100]. Lesion size (the thickness of infected footpad minus the thickness of uninfected footpad) was measured weekly using a Vernier caliper. Parasite numbers in the infected footpad were determined by the limiting dilution assay [101].

### Anisotropy assay

The plasma membrane fluidity of live *Leishmania* promastigotes was determined by measuring the fluorescence depolarization of TMA-DPH, as previously described for *T. brucei* [60]. Parasites were washed once with and resuspended in PBS at a density of 5.0×10^6^ cells/mL. TMA-DPH was added to a final concentration of 0.5 µM and allowed to stain the cell membrane for 20 min at 4°C, 25°C, 37°C, or 45°C in the dark. Anisotropic values were acquired using a T-mode Photon Technology International (Lawrenceville, NJ) C61/2000 spectrofluorimeter. Samples were excited at 358 nm, and emission was read at 430 nm, with 10-nm excitation and emission slit widths. Temperature was maintained by means of the PerkinElmer LS55 Biokinetics accessory. Data were corrected for light scattering with an unlabeled sample of cells, and anisotropy was calculated according to the equation r  =  (I_VV_ − GI_VH_)/(I_VV_ + 2GI_VH_), where r is the anisotropy value, I_VV_ is the emission intensity acquired with the excitation- and emission-polarizing filters set vertically, G is the instrument correction factor, and I_VH_ is the emission intensity acquired with the excitation-polarizing filter set vertically and the emission-polarizing filter set horizontally. Data points shown are the average of triplicate measurements with standard deviations.

### Statistical analysis

Most experiments (except for the Southern blot in Fig. 1) were repeated at least three times. The difference between two groups was determined by the Student's *t* test using Sigmaplot 11.0 (Systat Software Inc, San Jose, CA). *P* values indicating statistical significance were grouped into values of <0.05 and <0.01.

### List of accession numbers/ID numbers

- *Leishmania major* C14DM: LmjF11.1100 (TritrpDB)
- *Homo sapiens* C14DM: Q16850 (Genbank)
- *Aspergillus fumigatus* C14DM: XP_752137 (Genbank)
- *Candida albicans* C14DM: XP_716822 (Genbank)
- Mycobacterium tuberculosis C14DM: NP_215278 (Genbank)
- *Leishmania major* squalene epoxidase: LmjF.13.1620 (TritrpDB)
- *Leishmania major* squalene synthase: LmjF.31.2940 (TritrpDB)
- *Leishmania major* delta(24)-sterol C-methyltransferase: LmjF.36.2380 and LmjF.36.2390 (TritrpDB)

## Supporting Information

Figure S1 **Predicted sterol synthesis pathway in** ***Leishmania*** **from lanosterol to ergosterol.** I–XIII represent sterol intermediates or final products (formula weights in parentheses). I: lanosterol (426.7); II: 4,14-dimethyl-8,24-cholestadienol (412.7); III: 4-methyl-8,24-cholestadienol (398.6); IV: zymosterol (384.6); V: fecosterol (398.6); VI: cholesta-7,24-dienol (384.6); VII: episterol (398.6); VIII: cholesta-5,7,24-trienol (382.6); IX: 5-dehydroepisterol (396.6); X: ergosterol (396.6); XI: 14-methyl-zymosterol (398.6); XII: 14-methyl-fecosterol (412.6); XIII: cholesterol (386.6). C14DM: Sterol C14α-demethylase (the blue circle marks the C14-methyl group to be removed by C14DM). SMT: Sterol C24-methyl transferase. VIII, IX and X represent final sterol products synthesized by *Leishmania*; XIII (cholesterol, shaded) is salvaged from the host or environment; XI and XII (in red) represent the accumulated sterol intermediates when C14DM is blocked.
(PDF)

Figure S2 **Alignment of C14DMs from** ***L. major*** **(LmjF11.1100),** ***H. sapiens*** **(Q16850),** ***A. fumigatus*** **(XP_752137),** ***C. albicans*** **(XP_716822), and** ***M. tuberculosis*** **(NP_215278).** Highly conserved residues are highlighted in black. The asterisks mark the predicted sterol substrate binding site and the box indicates the predicted heme binding motif.
(PDF)

Figure S3 **The C14DM-GFP fusion protein is intact and functional.** (**A**) Log and day 3 stationary phase cell lysates from *c14dm* ^−^ and *c14dm* ^−^ */+C14DM-GFP* parasites were analyzed by Western blot, using antibodies against GFP (upper panel) or α-tubulin (lower panel). (**B**) Partial GC-MS chromatogram of lipids from *c14dm* ^−^ */+C14DM-GFP* promastigotes. VIII-X represent sterol species (shown in **Fig. S1** and **Fig. 4**) and their corresponding peaks are indicated by arrows.
(PDF)

Figure S4 **Accumulation of tetraploid cells in** ***c14dm*** **^−^ mutants.** Log phase (**A**–**C**) or stationary phase (**D**–**F**) promastigotes of WT (**A, D**), *c14dm* ^−^ (**B, E**), and *c14dm* ^−^ */+C14DM* (**C, F**) were fixed, permeablized and treated with RNase before staining with propidium iodide. Following flow cytometry, percentages of 1K1N and 2K2N cells are indicated. More 2K2N cells were observed in log phase when cells were more replicative.
(PDF)

Figure S5 **Sterol analysis in WT promastigotes.** Total lipids were extracted from WT promastigotes and analyzed by GC-MS. (**A**) Total ion current (TIC) chromatogram of lipids with m/z of 50–500. Std: internal standard (cholesta-3,5-diene). (**B**) Selected ion monitoring of sterol species with m/z of 396–397 (ergosterol and 5-dehydroepisterol). (**C**) Selected ion monitoring of sterol species with m/z of 398–399 (episterol). (**D**) Selected ion monitoring of sterol species with m/z of 386–387 (cholesterol). (**E**) Selected ion monitoring of sterol species with m/z of 382–383 (cholesta-5,7,24-trienol). (**F**) Selected ion monitoring of sterol species with m/z of 412–413 (14-methyl-fecosterol). Signal intensity (an arbitrary unit reflecting the abundance of the major ion) is indicated on the right.
(PDF)

Figure S6 **Sterol analysis in** ***c14dm*** **^−^ promastigotes.** Total lipids from *c14dm* ^−^ promastigotes were analyzed by GC-MS. (**A**) TIC chromatogram of lipids with m/z of 50–500. Std: internal standard (cholesta-3,5-diene). (**B**) Selected ion monitoring of sterol species with m/z of 396–397 (ergosterol and 5-dehydroepisterol). (**C**) Selected ion monitoring of sterol species with m/z of 398–399 (mainly14-methyl-zymosterol). (**D**) Selected ion monitoring of sterol species with m/z of 386–387 (cholesterol). (**E**) Selected ion monitoring of sterol species with m/z of 382–383 (cholesta-5,7,24-trienol). (**F**) Selected ion monitoring of sterol species with m/z of 412–413 (14-methyl-fecosterol). Signal intensity is indicated on the right.
(PDF)

Figure S7 **Sterol analysis in WT promastigotes treated with ITZ.** WT promastigotes were inoculated at 2.0×10^5^ cells/ml in the presence of ITZ (0.2 µM). Total lipids were extracted after three days and analyzed by GC-MS. (**A**) TIC chromatogram of lipids with m/z of 50–500. Std: internal standard (cholesta-3,5-diene). (**B**) Selected ion monitoring of sterol species with m/z of 396–397 (ergosterol and 5-dehydroepisterol). (**C**) Selected ion monitoring of sterol species with m/z of 398–399 (mainly14-methyl-zymosterol). (**D**) Selected ion monitoring of sterol species with m/z of 386–387 (cholesterol). (**E**) Selected ion monitoring of sterol species with m/z of 382–383 (cholesta-5,7,24-trienol). (**F**) Selected ion monitoring of sterol species with m/z of 412–413 (14-methyl-fecosterol). Signal intensity is indicated on the right.
(PDF)

Figure S8 **Sterol analysis in** ***c14dm*** **^−^** ***/+C14DM*** **promastigotes.** Total lipids were extracted from *c14dm* ^−^ */+C14DM* promastigotes and analyzed by GC-MS. (**A**) TIC chromatogram of lipids with m/z of 50–500. Std: internal standard (cholesta-3,5-diene). (**B**) Selected ion monitoring of sterol species with m/z of 396–397 (ergosterol and 5-dehydroepisterol). (**C**) Selected ion monitoring of sterol species with m/z of 398–399 (episterol). (**D**) Selected ion monitoring of sterol species with m/z of 386–387 (cholesterol). (**E**) Selected ion monitoring of sterol species with m/z of 382–383 (cholesta-5,7,24-trienol). (**F**) Selected ion monitoring of sterol species with m/z of 412–413 (14-methyl-fecosterol). Signal intensity is indicated on the right.
(PDF)

Figure S9 **Electron ionization mass spectra of** ***Leishmania*** **sterols.** Lipids from WT or *c14dm* ^−^ promastigotes were analyzed by GC-MS. Electron ionization spectra (70 eV) of sterol species based on retention time (RT) in Fig. S5–S8 were included. The predicted sterol types were indicated above each panel.
(PDF)

Figure S10 **Reduced LPG expression in** ***c14dm*** **^−^ mutants**. Log phase promastigotes of WT (**A**–**C**), *c14dm* ^−^ (**D–F**) and *c14dm* ^−^ */+C14DM* (**G–I**) were examined by immunofluorescence microscopy. **A**, **D**, and **G**: immune-staining with anti-LPG monoclonal antibody WIC 79.3, followed by a goat-anti-mouse IgG-FITC; **B**, **E**, and **H**: DNA staining using Hoechst 33242; **C**, **F**, and **I**: DIC images. (**J**–**L**) Log phase promastigotes of WT (**L**) or *c14dm* ^−^ (**K**) were labeled with anti-LPG antibody, followed by goat-anti-mouse IgG-FITC and then analyzed by flow cytometry. **L**: merge of **J** and **K**.
(PDF)

Figure S11 **Enhanced GP63 expression in log phase** ***c14dm*** **^−^ mutants.** Log phase promastigotes of WT (**A**–**C**), *c14dm* ^−^ (**D–F**) and *c14dm* ^−^ */+C14DM* (**G–I**) were examined by immunofluorescence microscopy. **A**, **D**, and **G**: immuno-staining with a monoclonal anti-GP63 antibody, followed by a goat-anti-mouse IgG-FITC; besides plasma membrane, GP63 was also found intracellularly; **B**, **E**, and **H**: DNA staining using Hoechst 33242; **C**, **F**, and **I**: DIC images.
(PDF)

Figure S12 ***C14dm*** **^−^ parasites survive poorly in murine macrophages (MΦs).** Purified metacyclics (black circle: WT; white circle: *c14dm* ^−^; black triangle: *c14dm* ^−^ */+C14DM*) were used to infect bone marrow MΦs from BALB/c mice. To show that MΦs possess microbicidal activity, WT parasites were also used to infect MΦs that were activated with 50 ng/ml of LPS and 50 ng/ml of IFN-γ (white triangle). Fraction of infected MΦs (**A**) and number of parasites per 100 MΦs (**B**) were recorded. Error bars represent standard deviations from triplicates.
(PDF)

Figure S13 **Isolated lesion amastigotes.** *L. major* WT amastigotes were isolated from infected BALB/c mice (footpads) and subjected to fluorescence microscopy. (**A**) DIC image; (**B**) DNA staining; (**C**) merge of **A** and **B**. Arrows indicate amastigotes and arrowheads indicate mouse cells/debris.
(PDF)

Figure S14 **Analysis of sterols in WT amastigotes.** Total lipids from WT lesion amastigotes were analyzed by GC-MS. (**A**) TIC chromatogram of lipids with m/z of 50–500. Std: internal standard (cholesta-3,5-diene). (**B**) Selected ion monitoring of sterol species with m/z of 386–387 (cholesterol). (**C**) Selected ion monitoring of sterol species with m/z of 396–397 (ergosterol and 5-dehydroepisterol). (**D**) Selected ion monitoring of sterol species with m/z of 398–399 (episterol). (**E**) Selected ion monitoring of sterol species with m/z of 412–413 (14-methyl-fecosterol). Retention times for major peaks are marked in **A**–**E**. Signal intensity is indicated in each panel.
(PDF)

Figure S15 **Analysis of sterols in** ***c14dm*** **^−^ amastigotes.** Total lipids from *c14dm* ^−^ amastigotes were analyzed by GC-MS. (**A**) TIC chromatogram of lipids with m/z of 50–500. Std: internal standard (cholesta-3,5-diene). (**B**) Selected ion monitoring of sterol species with m/z of 386–387 (cholesterol). (**C**) Selected ion monitoring of sterol species with m/z of 396–397 (ergosterol and 5-dehydroepisterol). (**D**) Selected ion monitoring of sterol species with m/z of 398–399 (mainly14-methyl-zymosterol). (**E**) Selected ion monitoring of sterol species with m/z of 412–413 (14-methyl-fecosterol). Retention times for major peaks are marked in **A**–**E**. Signal intensity is indicated in each panel.
(PDF)

Figure S16 **Analysis of sterols in uninfected mouse tissue.** Total lipids from uninfected mouse footpads were analyzed by GC-MS. (**A**) TIC chromatogram of lipids with m/z of 50–500. Std: internal standard (cholesta-3,5-diene). (**B**) Selected ion monitoring of sterol species with m/z of 386–387 (cholesterol). (**C**) Selected ion monitoring of sterol species with m/z of 396–397 (ergosterol and 5-dehydroepisterol). (**D**) Selected ion monitoring of sterol species with m/z of 398–399 (episterol). (**E**) Selected ion monitoring of sterol species with m/z of 412–413 (14-methyl-fecosterol). Retention times for major peaks are marked in **A**–**E**. Signal intensity is indicated in each panel.
(PDF)

Figure S17 **Analysis of sterols in WT promastigotes.** Total lipids from WT promastigotes were analyzed by GC-MS. (**A**) TIC chromatogram of lipids with m/z of 50–500. Std: internal standard (cholesta-3,5-diene). (**B**)–(**F**) Selected ion monitoring of sterol species with m/z of 386–387 (**B**), 396–397 (**C**), 398–399 (**D**), 412–413 (**E**), and 382–383 (**F**). Retention times for major peaks are marked in **A**–**E**. Signal intensity is indicated in each panel.
(PDF)

Figure S18 **Analysis of sterols in** ***c14dm*** **^−^ promastigotes.** Total lipids from *c14dm* ^−^ promastigotes were analyzed by GC-MS. (**A**) TIC chromatogram of lipids with m/z of 50–500. Std: internal standard (cholesta-3,5-diene). (**B**)–(**F**) Selected ion monitoring of sterol species with m/z of 386–387 (**B**), 396–397 (**C**), 398–399 (**D**), 412–413 (**E**), and 382–383 (**F**). Retention times for major peaks are marked in **A**–**E**. Signal intensity is indicated in each panel.
(PDF)

Figure S19 **ITZ treatment leads to extreme sensitivity to heat in several** ***Leishmania*** **species.** (**A**) Log phase promastigotes of *L. major* LV39 (black circle), *L. amazonensis* (white circle), *L. mexicana* (black triangle) and *L. donovani* (white triangle) were cultured in various concentrations of ITZ. Culture densities were determined after 48 hours. (**B**) Promastigotes were grown in the absence or presence of ITZ (at IC25 concentrations) to stationary phase. Cells were then incubated at 37°C/5% CO_2_ and cell viability was measured after 8 hours (black bars: *L. major* LV39, white bars: *L. amazonensis*, dark grey bars: *L. mexicana*, light grey bar: *L. donovani*). Experiments were repeated three times and error bars represent standard deviations.
(PDF)

Figure S20 **Ability of** ***c14dm*** **^−^ mutants to survive nitrosative, oxidative and acidic pH stress.** Stationary phase promastigotes of WT (black circle, black bars), *c14dm* ^−^ (white circle, white bars), and *c14dm* ^−^ */+c14dm* (black triangle, grey bars) were treated with various concentrations of SNAP (**A–B**), H_2_O_2_ (**C–D**), or incubated in pH 5.0 media (**E–F**). Cell density and percentage of dead cells were measured after 48 hours. Experiments were repeated three times and error bars represent standard deviations.
(PDF)

Table S1 **Sterol composition in** ***Leishmania*** **promastigotes.** Promastigotes were cultured in the absence or presence of ITZ (provided at IC25 concentrations: 120 nM for *L. major*, 81 nM for *L. donovani*, 3.3 nM for *L. mexicana*, and 25 nM for *L. amazonensis*) from early log phase to day 1 stationary phase and total lipids were analyzed by GC-MS. Abundances of ergosterol, 5-dehydroepisterol, cholesterol, cholesta-5,7,24-trienol, 14-methyl sterols (14-methylfecosterol + 14-methylzymosterol), and total sterols were estimated in relation to the internal standard cholesta-3,5-diene (provided at 2.0×10^7^ molecules/cell). Analyses were repeated 3 times and averaged values ± standard deviations (SDs) were shown. ND: not detectable.
(PDF)

Table S2 **Susceptibility of** ***Leishmania*** **parasites to ITZ (IC values in µM).** Promastigotes were inoculated at 2.0×10^5^ cells/ml in various concentrations of ITZ and culture densities were determined after 48 hours. IC25, IC50, and IC90 are ITZ concentrations that inhibit growth by 25%, 50%, and 90%, respectively, in comparison to control cultures (no ITZ). Experiments were performed three times (average ± SD).
(PDF)

Table S3 **List of oligonucleotides used in this study.** Sequences in lowercase represent restriction enzyme recognition sites.
(PDF)
