# Supplementary figures and images for: Sterol Biosynthesis Is Required for Heat Resistance but Not Extracellular Survival in Leishmania
Source: PLoS Pathog. 2014 Oct 23;10(10):e1004427. doi: 10.1371/journal.ppat.1004427 (PMC4207814; doi:10.1371/journal.ppat.1004427)

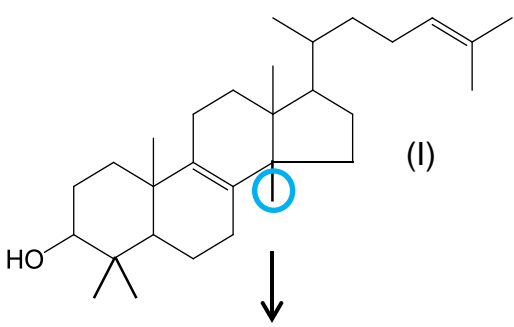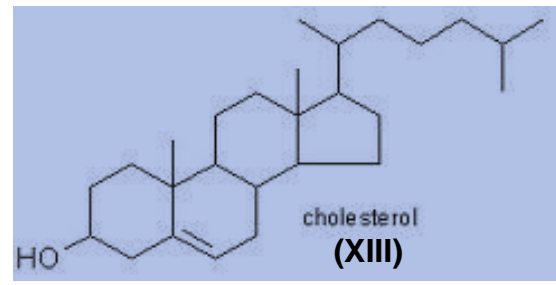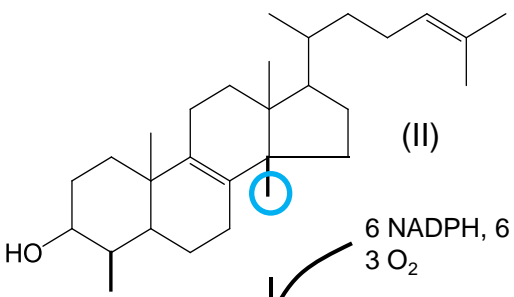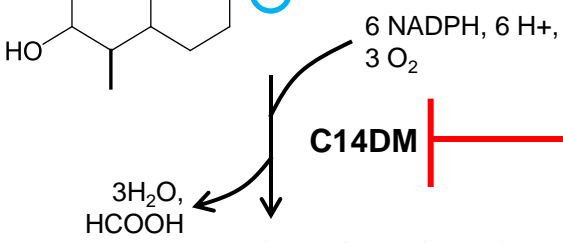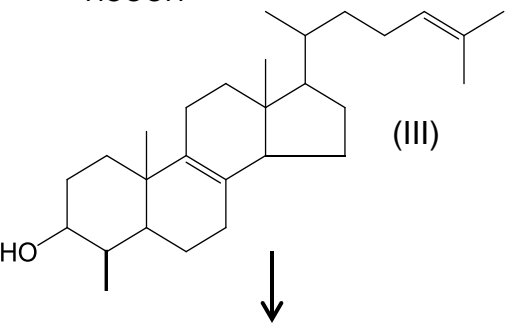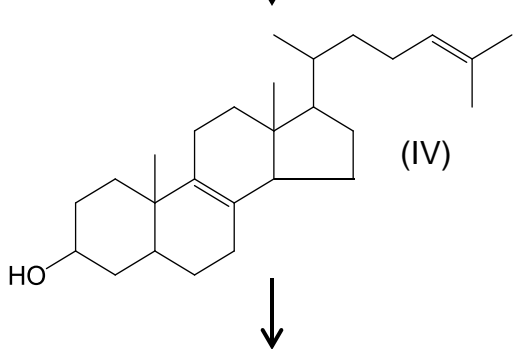

**SMT**

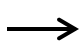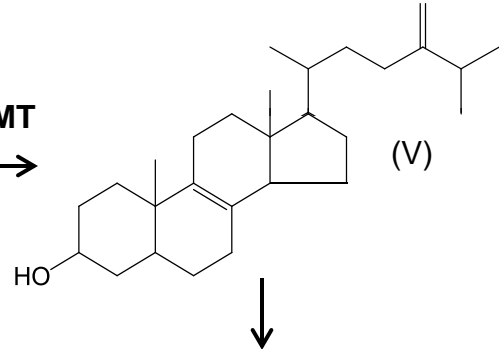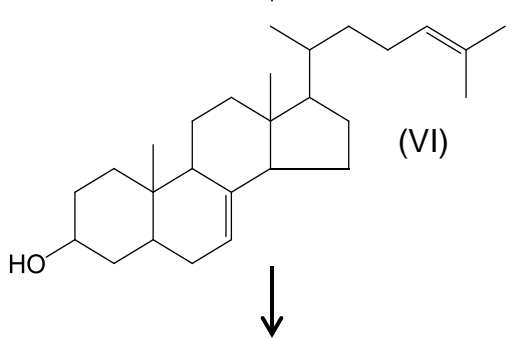

**SMT**

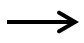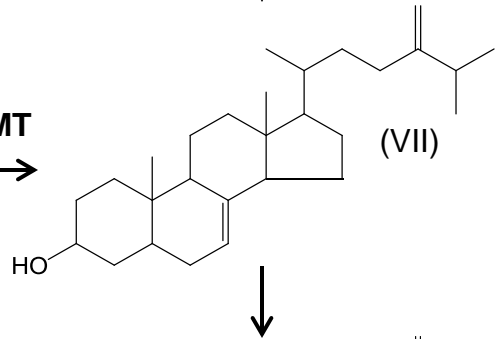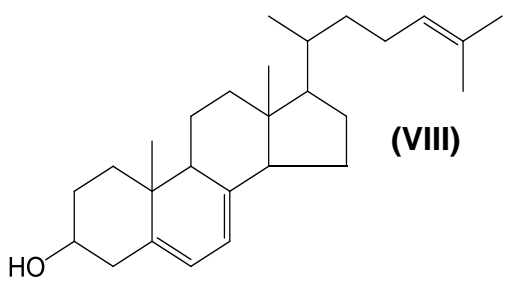

**SMT**

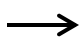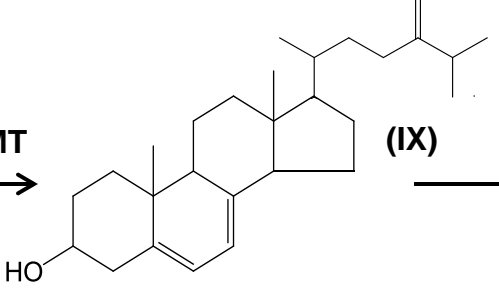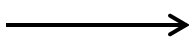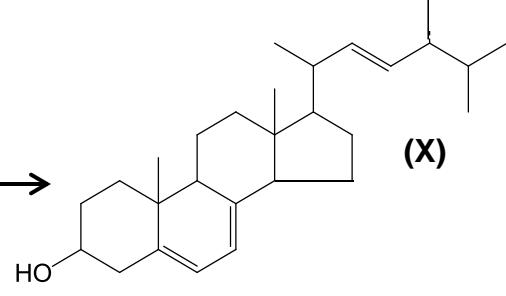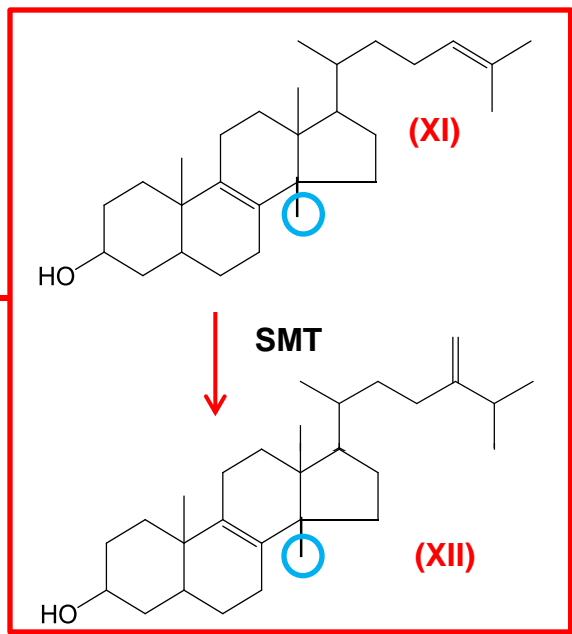

Supplement: Figure S1 — Predicted sterol synthesis pathway in Leishmania from lanosterol to ergosterol. I–XIII represent sterol intermediates or final products (formula weights in parentheses). I: lanosterol (426.7); II: 4,14-dimethyl-8,24-cholestadienol (412.7); III: 4-methyl-8,24-cholestadienol (398.6); IV: zymosterol (384.6); V: fecosterol (398.6); VI: cholesta-7,24-dienol (384.6); VII: episterol (398.6); VIII: cholesta-5,7,24-trienol (382.6); IX: 5-dehydroepisterol (396.6); X: ergosterol (396.6); XI: 14-methyl-zymosterol (398.6); XII: 14-methyl-fecosterol (412.6); XIII: cholesterol (386.6). C14DM: Sterol C14α-demethylase (the blue circle marks the C14-methyl group to be removed by C14DM). SMT: Sterol C24-methyl transferase. VIII, IX and X represent final sterol products synthesized by Leishmania; XIII (cholesterol, shaded) is salvaged from the host or environment; XI and XII (in red) represent the accumulated sterol intermediates when C14DM is blocked. (PDF) [file ppat.1004427.s001.pdf]

**A**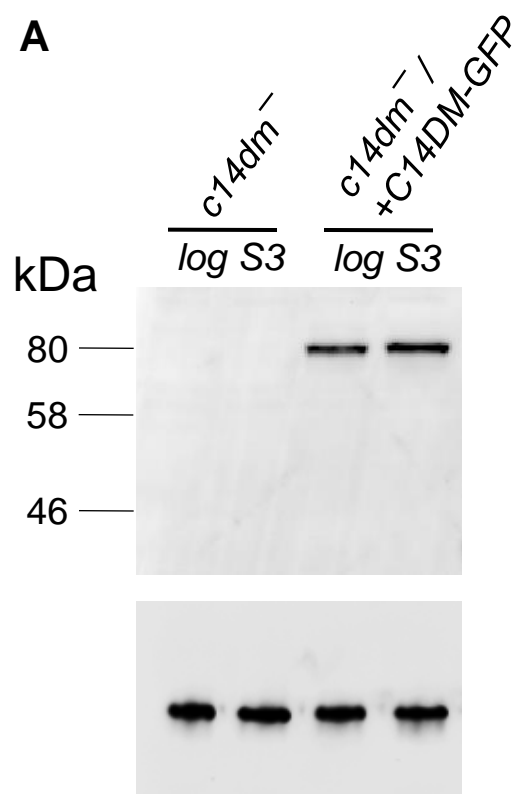**B**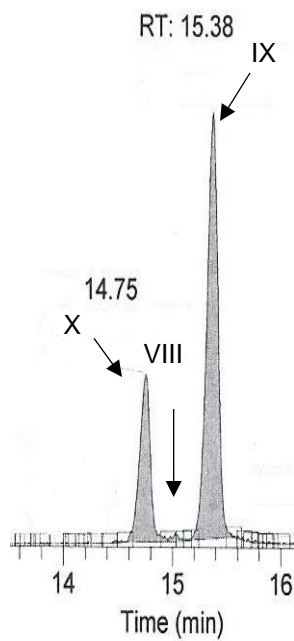

Supplement: Figure S3 — The C14DM-GFP fusion protein is intact and functional. (A) Log and day 3 stationary phase cell lysates from c14dm − and c14dm − /+C14DM-GFP parasites were analyzed by Western blot, using antibodies against GFP (upper panel) or α-tubulin (lower panel). (B) Partial GC-MS chromatogram of lipids from c14dm − /+C14DM-GFP promastigotes. VIII-X represent sterol species (shown in Fig. S1 and Fig. 4 ) and their corresponding peaks are indicated by arrows. (PDF) [file ppat.1004427.s003.pdf]

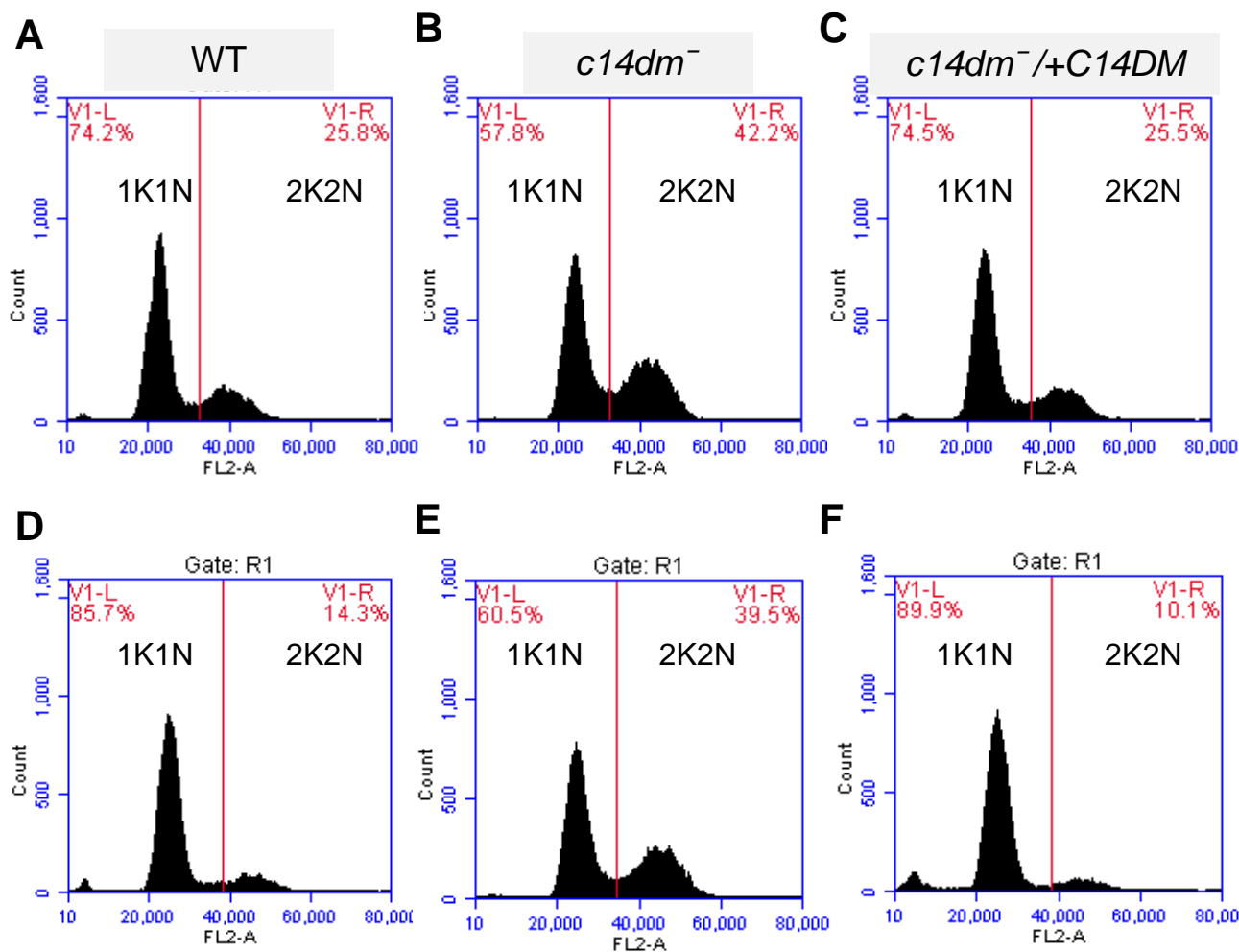

Supplement: Figure S4 — Accumulation of tetraploid cells in c14dm − mutants. Log phase (A–C) or stationary phase (D–F) promastigotes of WT (A, D), c14dm − (B, E), and c14dm − /+C14DM (C, F) were fixed, permeablized and treated with RNase before staining with propidium iodide. Following flow cytometry, percentages of 1K1N and 2K2N cells are indicated. More 2K2N cells were observed in log phase when cells were more replicative. (PDF) [file ppat.1004427.s004.pdf]

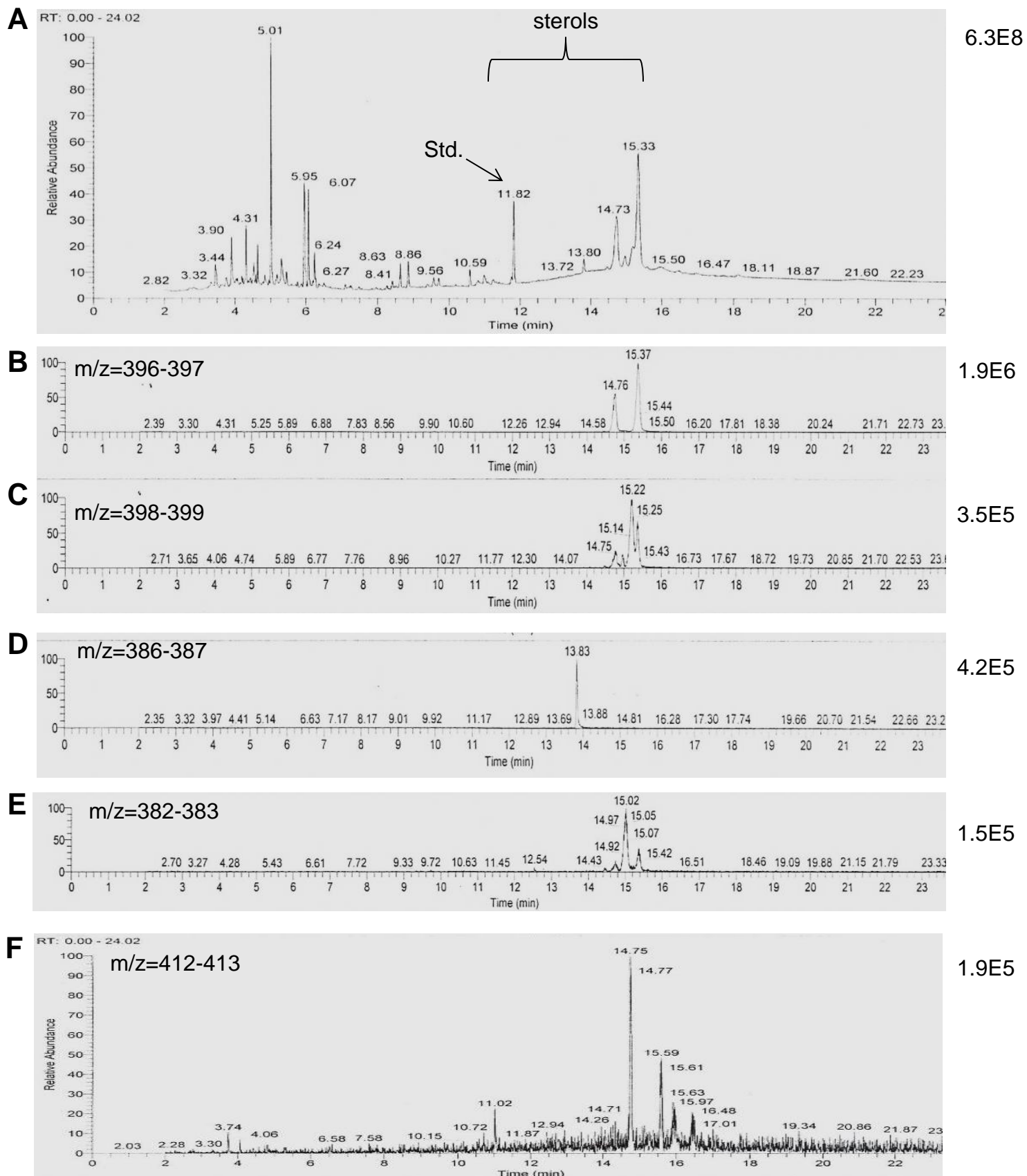

Supplement: Figure S5 — Sterol analysis in WT promastigotes. Total lipids were extracted from WT promastigotes and analyzed by GC-MS. (A) Total ion current (TIC) chromatogram of lipids with m/z of 50–500. Std: internal standard (cholesta-3,5-diene). (B) Selected ion monitoring of sterol species with m/z of 396–397 (ergosterol and 5-dehydroepisterol). (C) Selected ion monitoring of sterol species with m/z of 398–399 (episterol). (D) Selected ion monitoring of sterol species with m/z of 386–387 (cholesterol). (E) Selected ion monitoring of sterol species with m/z of 382–383 (cholesta-5,7,24-trienol). (F) Selected ion monitoring of sterol species with m/z of 412–413 (14-methyl-fecosterol). Signal intensity (an arbitrary unit reflecting the abundance of the major ion) is indicated on the right. (PDF) [file ppat.1004427.s005.pdf]

**A**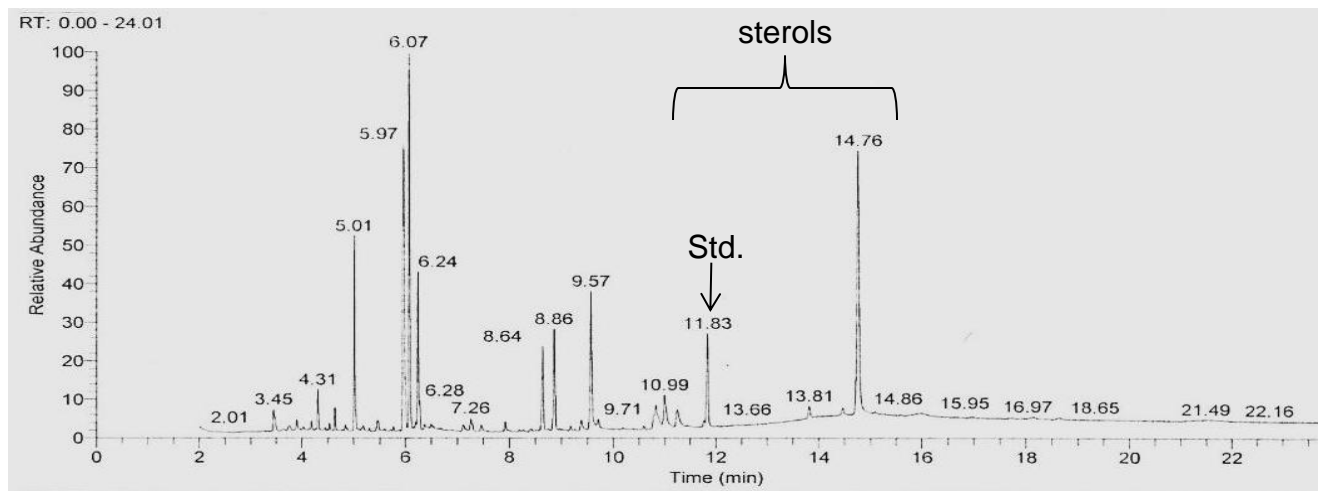

8.6E8

**B**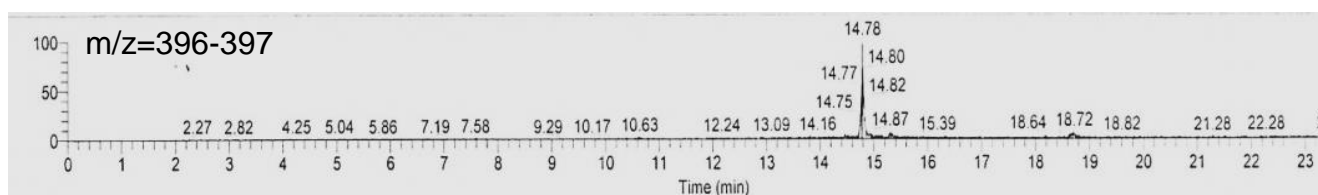

1.7E5

**C**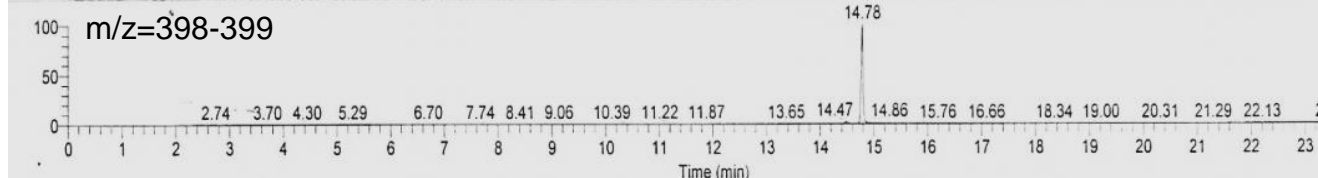

3.8E6

**D**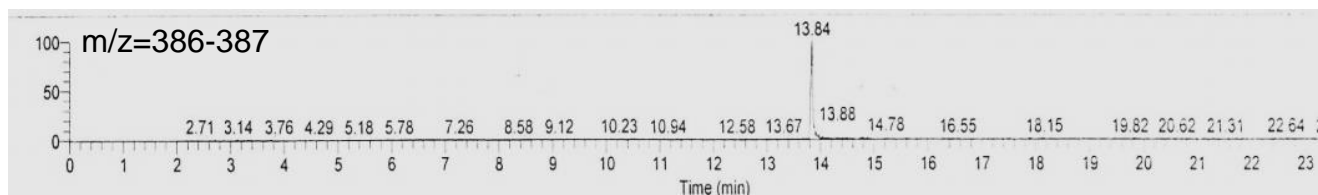

2.6E5

**E**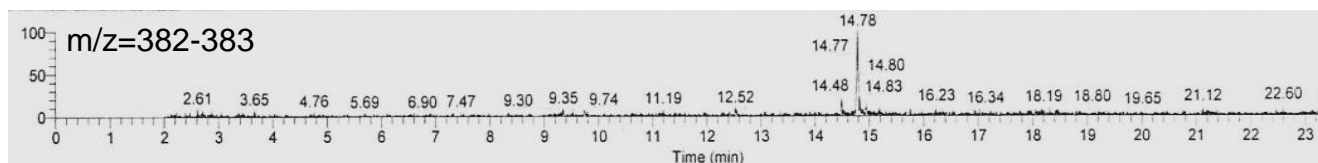

3.2E4

**F**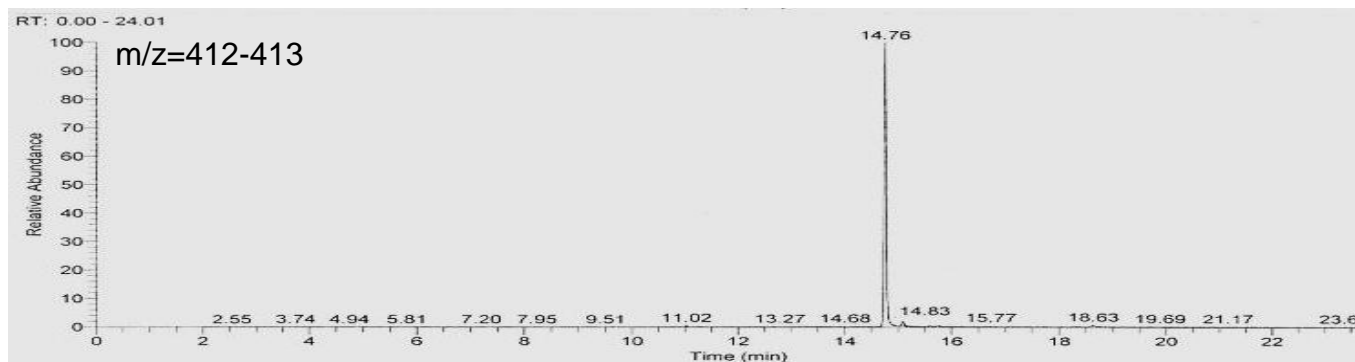

6.3E6

Supplement: Figure S6 — Sterol analysis in c14dm − promastigotes. Total lipids from c14dm − promastigotes were analyzed by GC-MS. (A) TIC chromatogram of lipids with m/z of 50–500. Std: internal standard (cholesta-3,5-diene). (B) Selected ion monitoring of sterol species with m/z of 396–397 (ergosterol and 5-dehydroepisterol). (C) Selected ion monitoring of sterol species with m/z of 398–399 (mainly14-methyl-zymosterol). (D) Selected ion monitoring of sterol species with m/z of 386–387 (cholesterol). (E) Selected ion monitoring of sterol species with m/z of 382–383 (cholesta-5,7,24-trienol). (F) Selected ion monitoring of sterol species with m/z of 412–413 (14-methyl-fecosterol). Signal intensity is indicated on the right. (PDF) [file ppat.1004427.s006.pdf]

**A**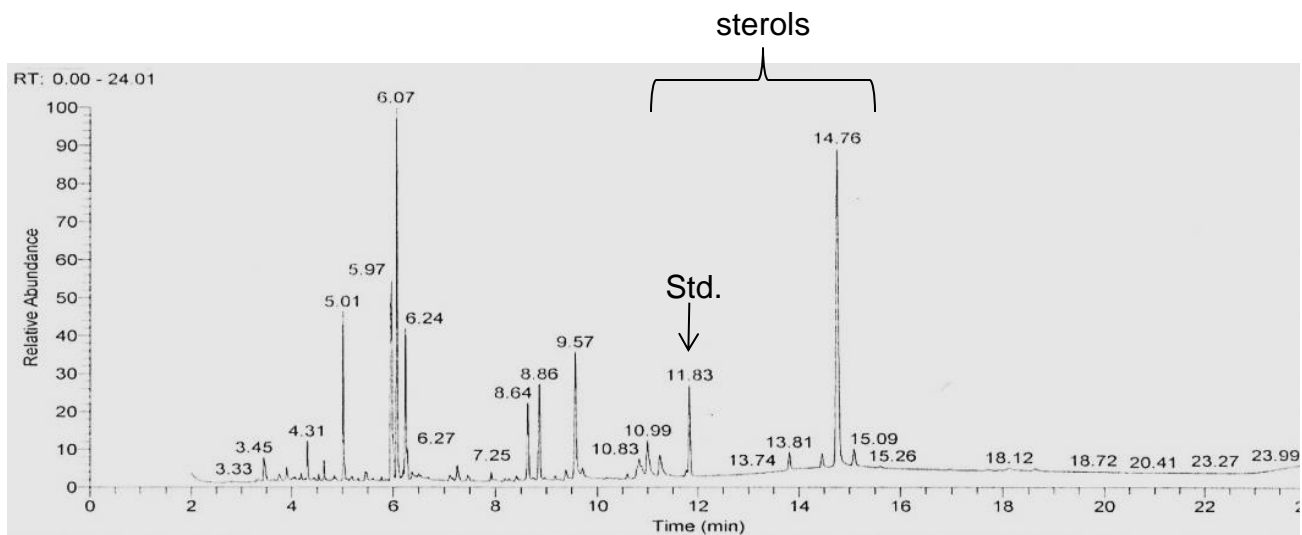**B**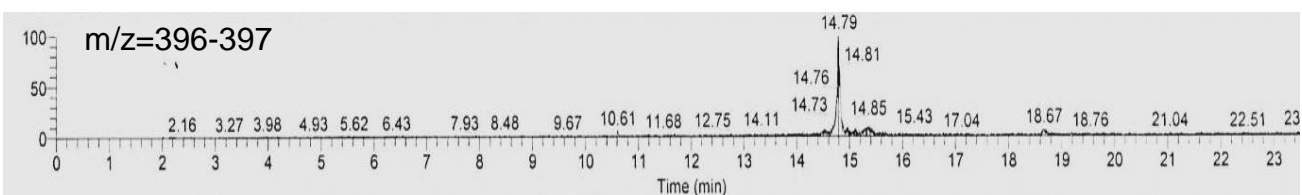**C**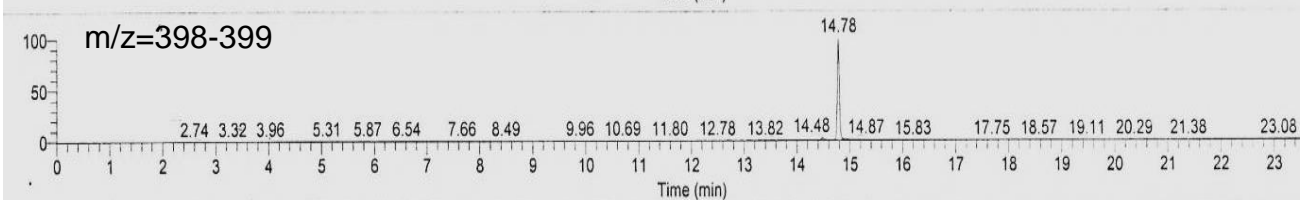**D**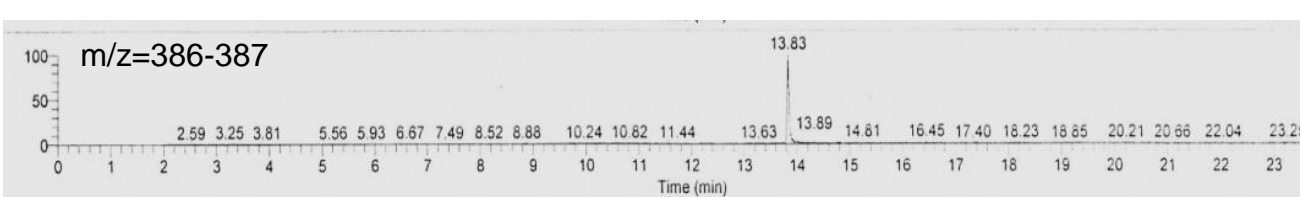**E**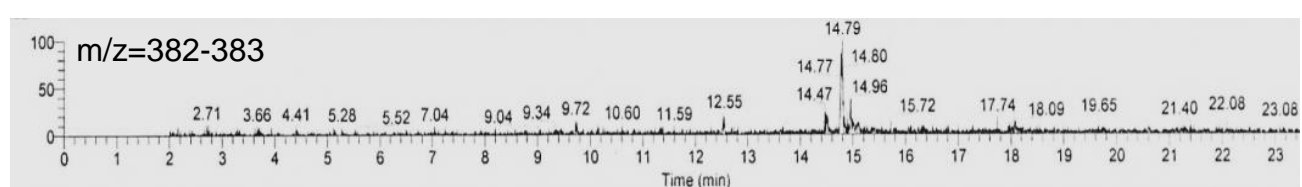**F**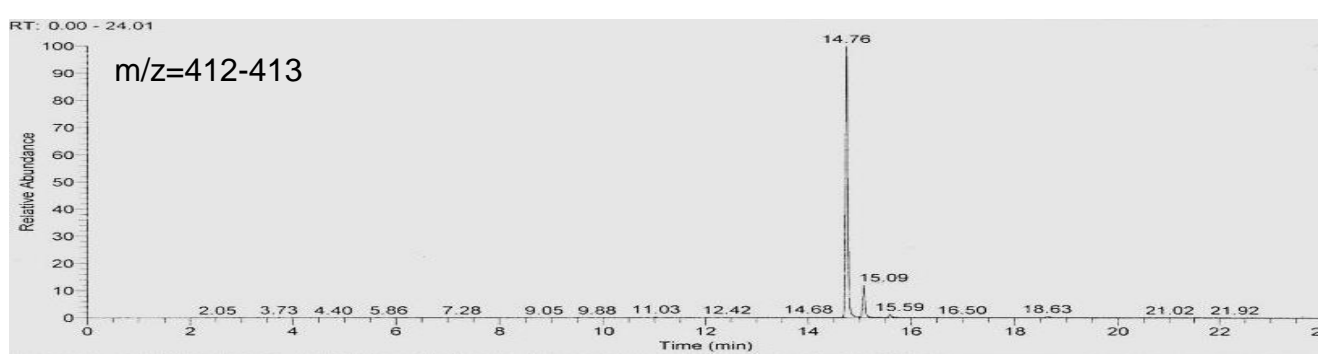

Supplement: Figure S7 — Sterol analysis in WT promastigotes treated with ITZ. WT promastigotes were inoculated at 2.0×10^5^ cells/ml in the presence of ITZ (0.2 µM). Total lipids were extracted after three days and analyzed by GC-MS. (A) TIC chromatogram of lipids with m/z of 50–500. Std: internal standard (cholesta-3,5-diene). (B) Selected ion monitoring of sterol species with m/z of 396–397 (ergosterol and 5-dehydroepisterol). (C) Selected ion monitoring of sterol species with m/z of 398–399 (mainly14-methyl-zymosterol). (D) Selected ion monitoring of sterol species with m/z of 386–387 (cholesterol). (E) Selected ion monitoring of sterol species with m/z of 382–383 (cholesta-5,7,24-trienol). (F) Selected ion monitoring of sterol species with m/z of 412–413 (14-methyl-fecosterol). Signal intensity is indicated on the right. (PDF) [file ppat.1004427.s007.pdf]

**A**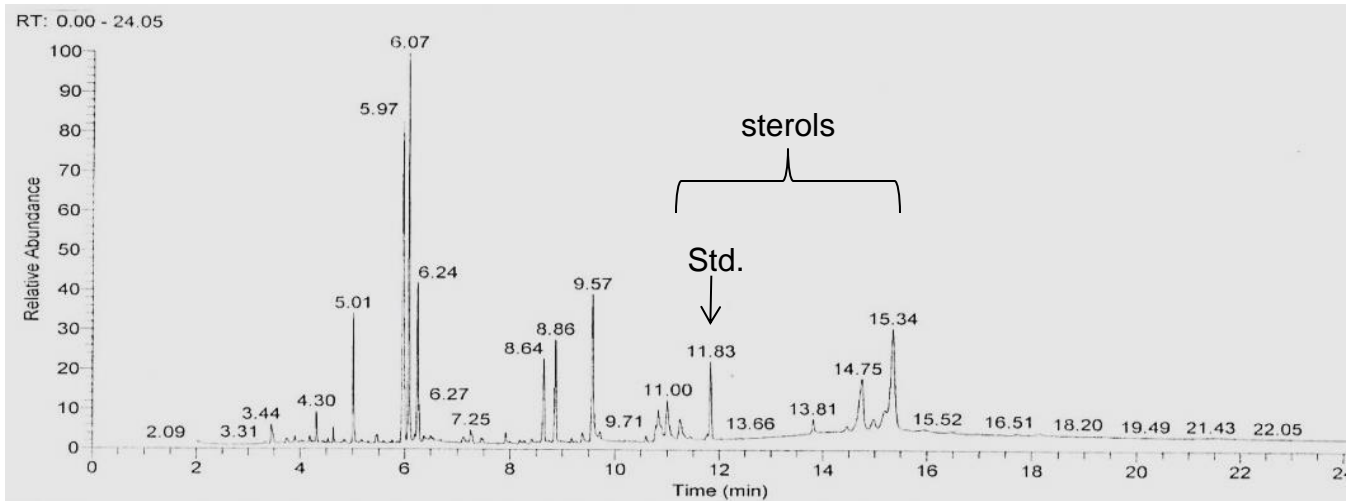

1.2E9

**B**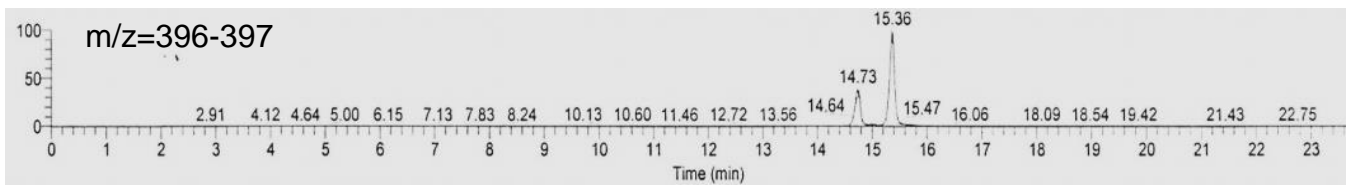

1.4E6

**C**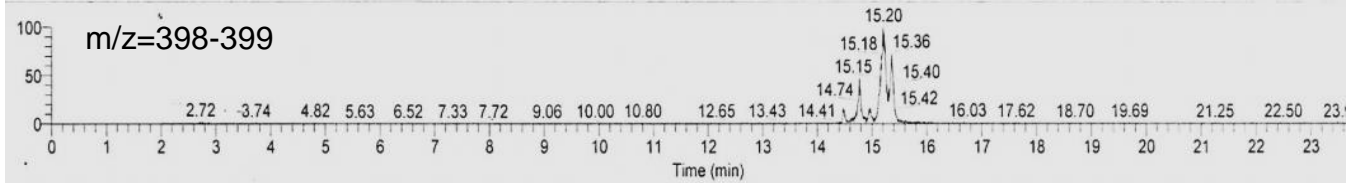

2.6E5

**D**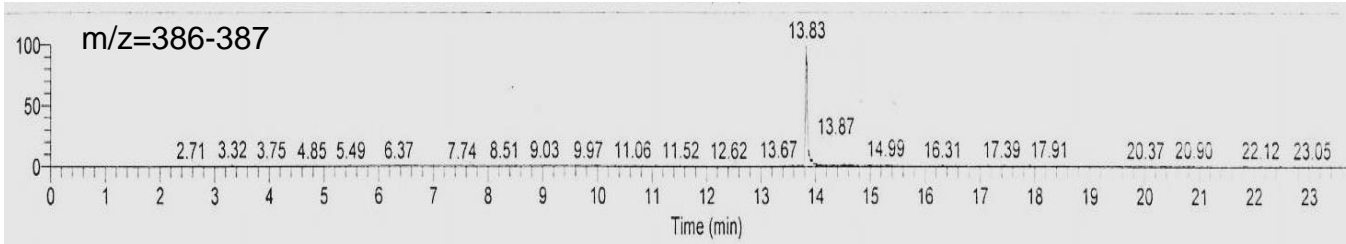

3.2E5

**E**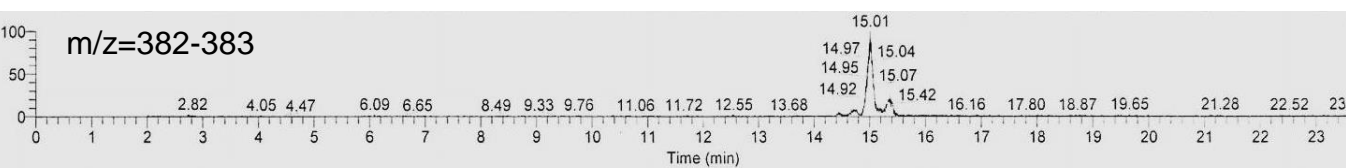

1.6E5

**F**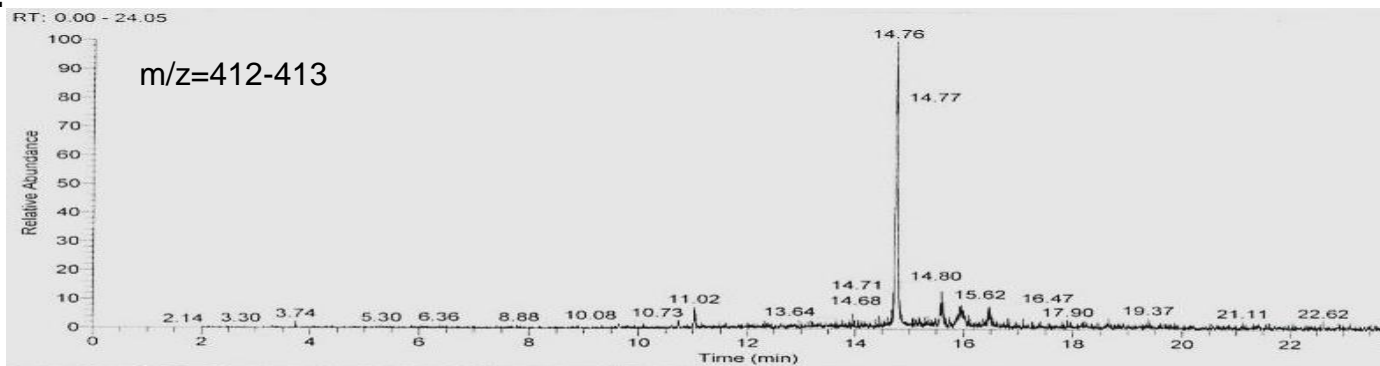

5.9E5

Supplement: Figure S8 — Sterol analysis in c14dm − /+C14DM promastigotes. Total lipids were extracted from c14dm − /+C14DM promastigotes and analyzed by GC-MS. (A) TIC chromatogram of lipids with m/z of 50–500. Std: internal standard (cholesta-3,5-diene). (B) Selected ion monitoring of sterol species with m/z of 396–397 (ergosterol and 5-dehydroepisterol). (C) Selected ion monitoring of sterol species with m/z of 398–399 (episterol). (D) Selected ion monitoring of sterol species with m/z of 386–387 (cholesterol). (E) Selected ion monitoring of sterol species with m/z of 382–383 (cholesta-5,7,24-trienol). (F) Selected ion monitoring of sterol species with m/z of 412–413 (14-methyl-fecosterol). Signal intensity is indicated on the right. (PDF) [file ppat.1004427.s008.pdf]

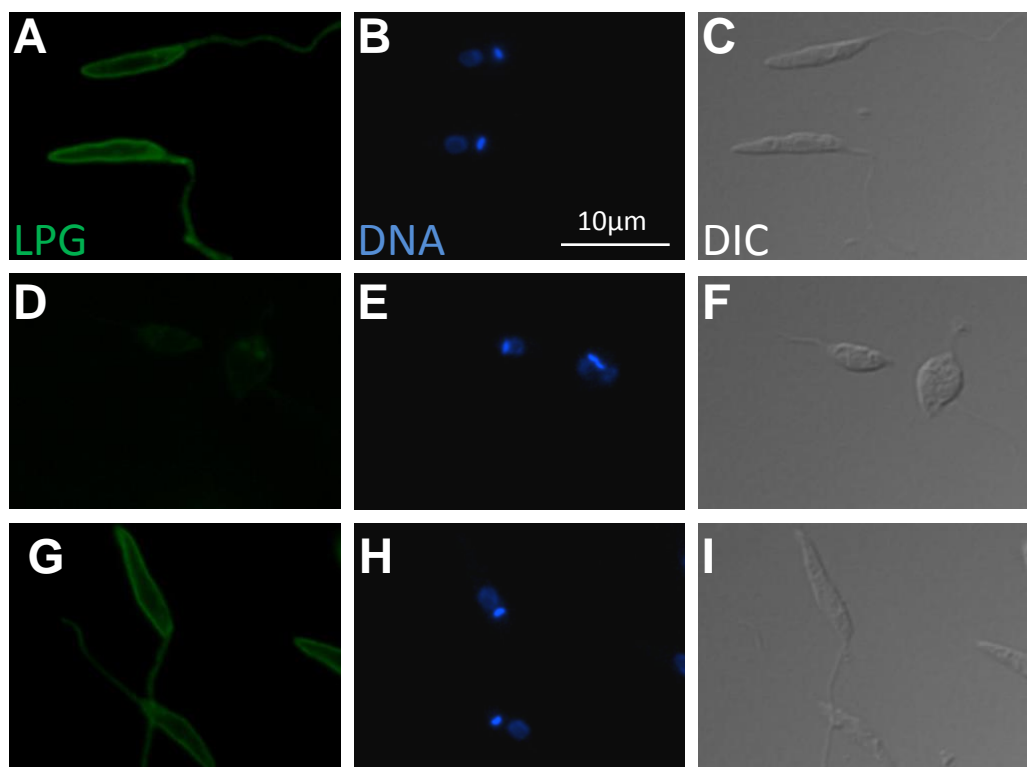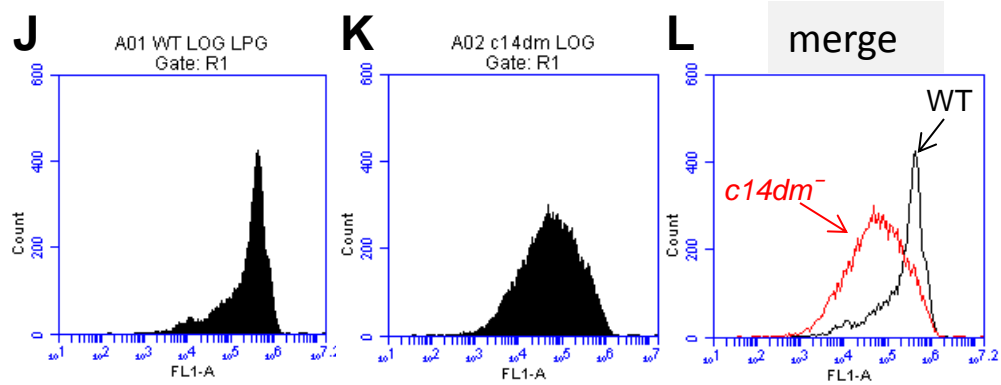

Supplement: Figure S10 — Reduced LPG expression in c14dm − mutants. Log phase promastigotes of WT (A–C), c14dm − (D–F) and c14dm − /+C14DM (G–I) were examined by immunofluorescence microscopy. A, D, and G: immune-staining with anti-LPG monoclonal antibody WIC 79.3, followed by a goat-anti-mouse IgG-FITC; B, E, and H: DNA staining using Hoechst 33242; C, F, and I: DIC images. (J–L) Log phase promastigotes of WT (L) or c14dm − (K) were labeled with anti-LPG antibody, followed by goat-anti-mouse IgG-FITC and then analyzed by flow cytometry. L: merge of J and K. (PDF) [file ppat.1004427.s010.pdf]

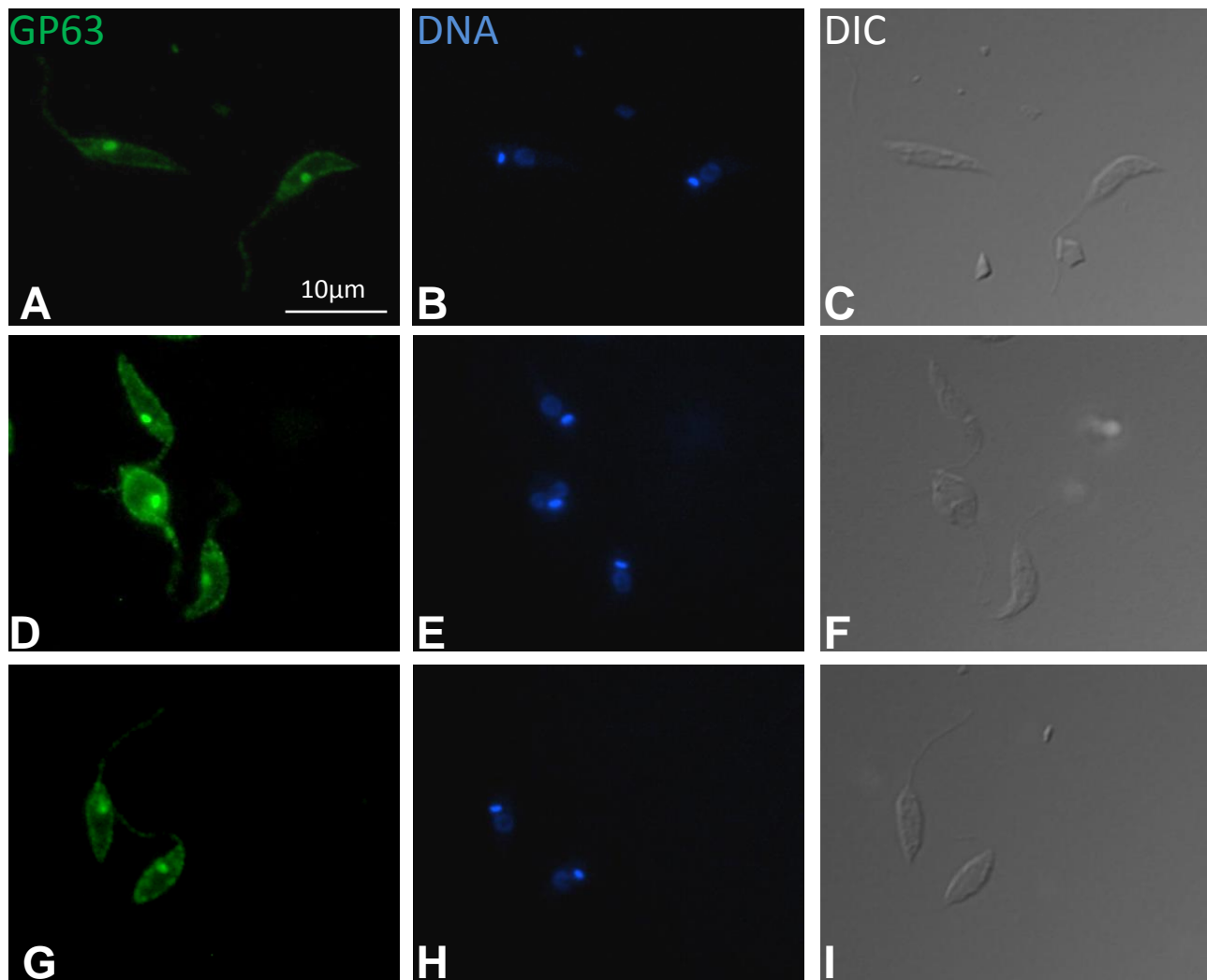

Supplement: Figure S11 — Enhanced GP63 expression in log phase c14dm − mutants. Log phase promastigotes of WT (A–C), c14dm − (D–F) and c14dm − /+C14DM (G–I) were examined by immunofluorescence microscopy. A, D, and G: immuno-staining with a monoclonal anti-GP63 antibody, followed by a goat-anti-mouse IgG-FITC; besides plasma membrane, GP63 was also found intracellularly; B, E, and H: DNA staining using Hoechst 33242; C, F, and I: DIC images. (PDF) [file ppat.1004427.s011.pdf]

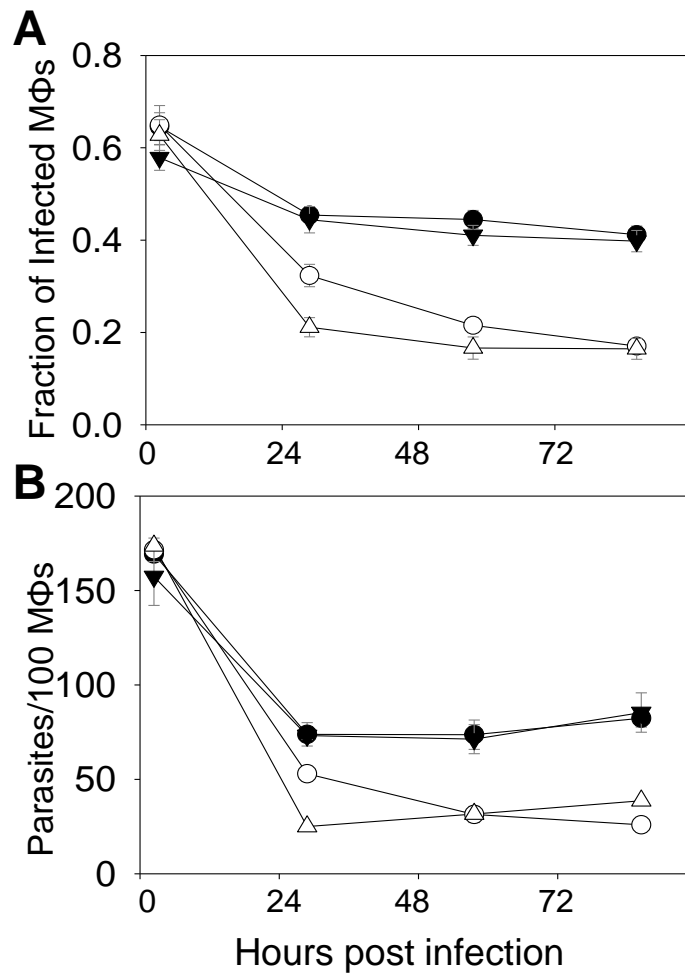

Supplement: Figure S12 — C14dm − parasites survive poorly in murine macrophages (MΦs). Purified metacyclics (black circle: WT; white circle: c14dm −; black triangle: c14dm − /+C14DM) were used to infect bone marrow MΦs from BALB/c mice. To show that MΦs possess microbicidal activity, WT parasites were also used to infect MΦs that were activated with 50 ng/ml of LPS and 50 ng/ml of IFN-γ (white triangle). Fraction of infected MΦs (A) and number of parasites per 100 MΦs (B) were recorded. Error bars represent standard deviations from triplicates. (PDF) [file ppat.1004427.s012.pdf]

**A**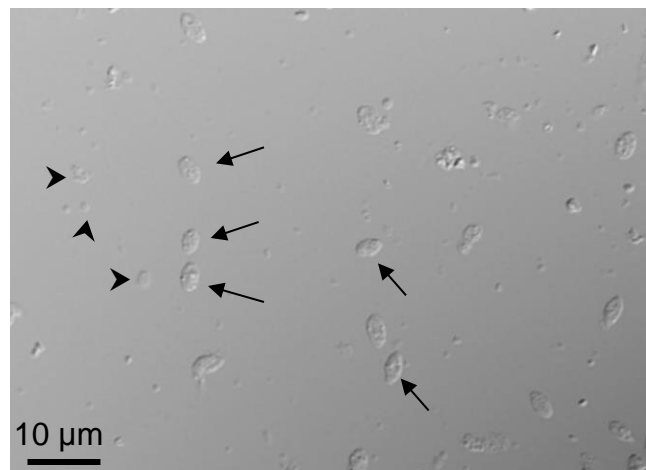**B**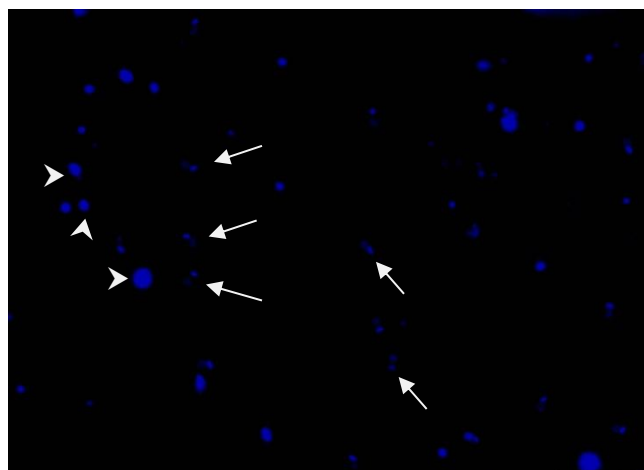**C**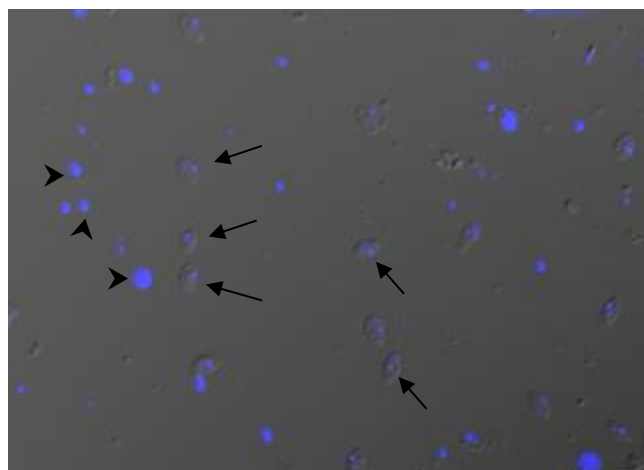

Supplement: Figure S13 — Isolated lesion amastigotes. L. major WT amastigotes were isolated from infected BALB/c mice (footpads) and subjected to fluorescence microscopy. (A) DIC image; (B) DNA staining; (C) merge of A and B. Arrows indicate amastigotes and arrowheads indicate mouse cells/debris. (PDF) [file ppat.1004427.s013.pdf]

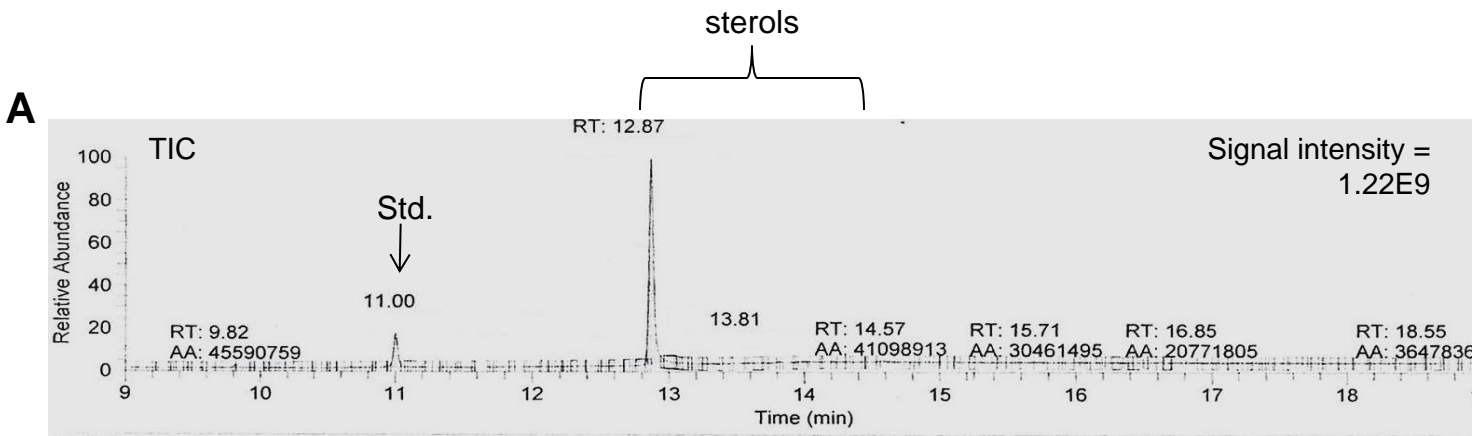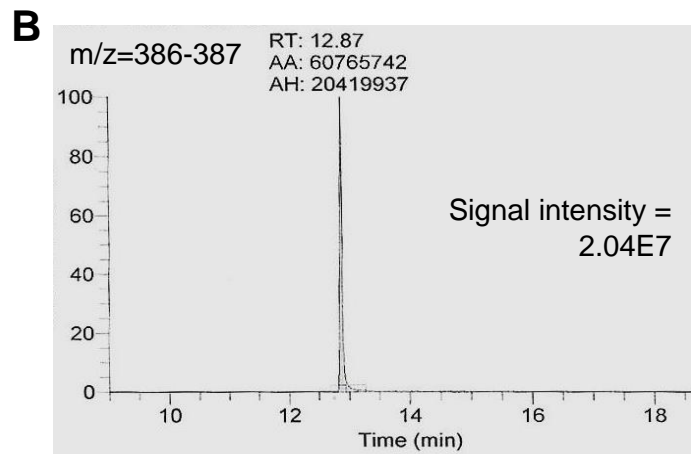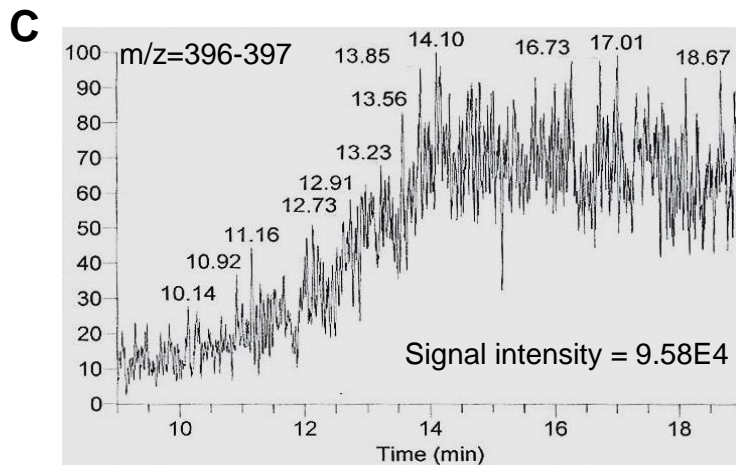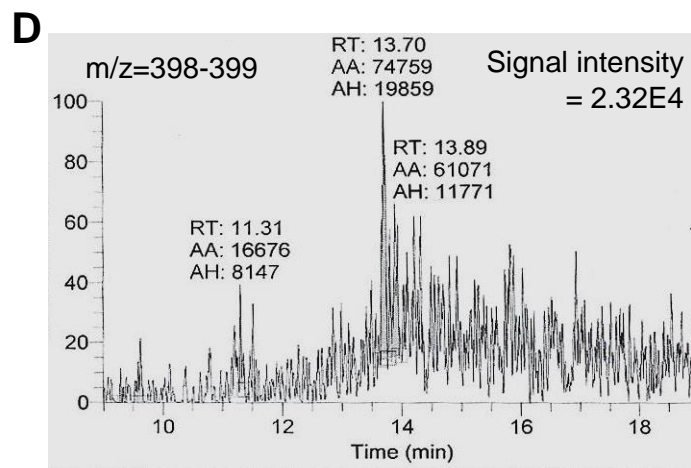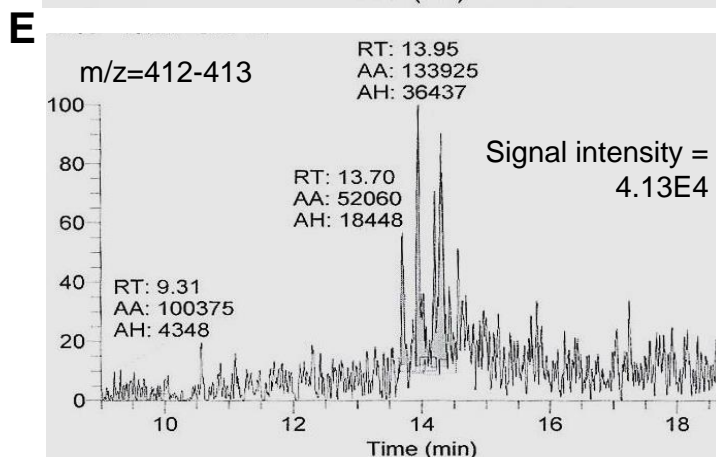

Supplement: Figure S14 — Analysis of sterols in WT amastigotes. Total lipids from WT lesion amastigotes were analyzed by GC-MS. (A) TIC chromatogram of lipids with m/z of 50–500. Std: internal standard (cholesta-3,5-diene). (B) Selected ion monitoring of sterol species with m/z of 386–387 (cholesterol). (C) Selected ion monitoring of sterol species with m/z of 396–397 (ergosterol and 5-dehydroepisterol). (D) Selected ion monitoring of sterol species with m/z of 398–399 (episterol). (E) Selected ion monitoring of sterol species with m/z of 412–413 (14-methyl-fecosterol). Retention times for major peaks are marked in A–E. Signal intensity is indicated in each panel. (PDF) [file ppat.1004427.s014.pdf]

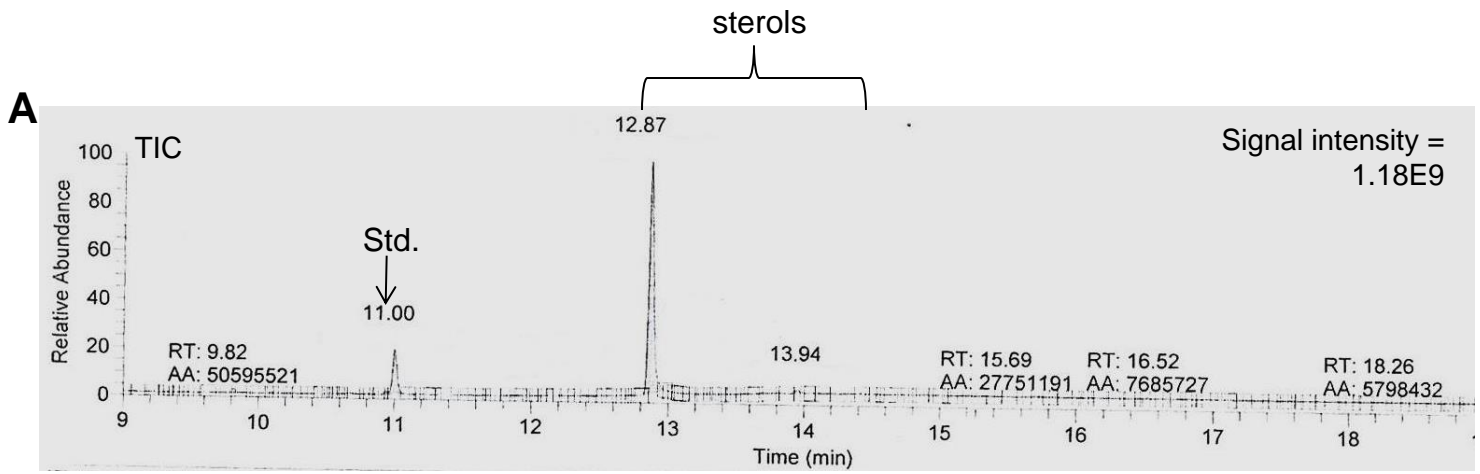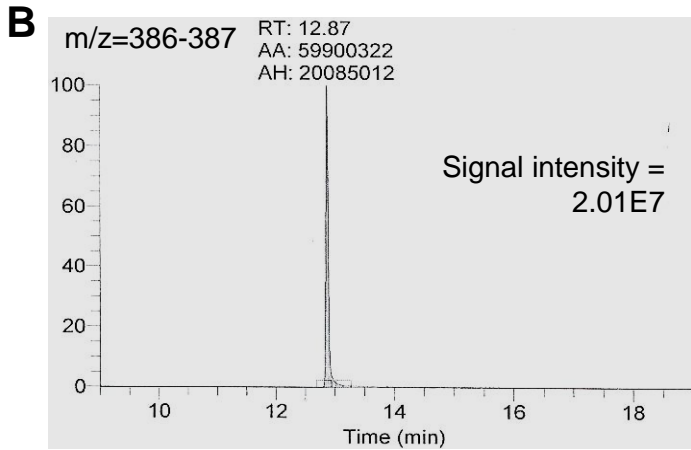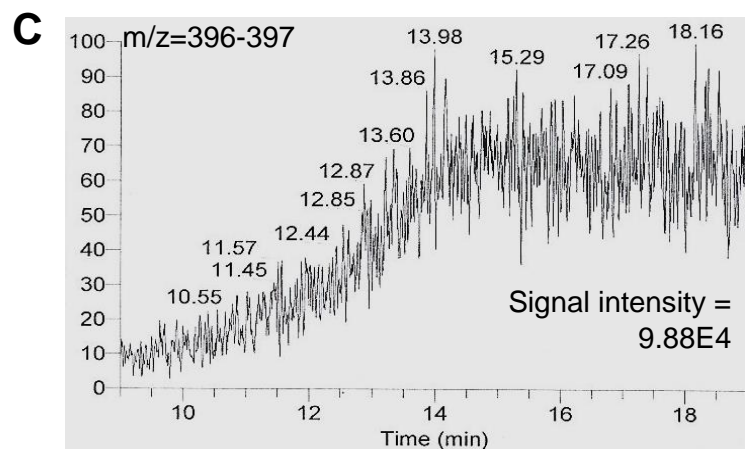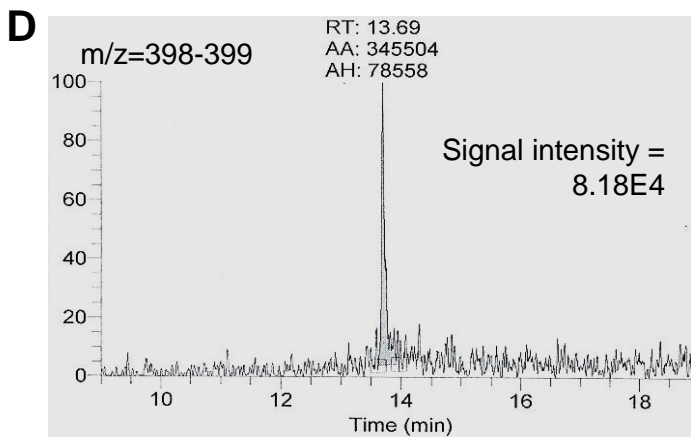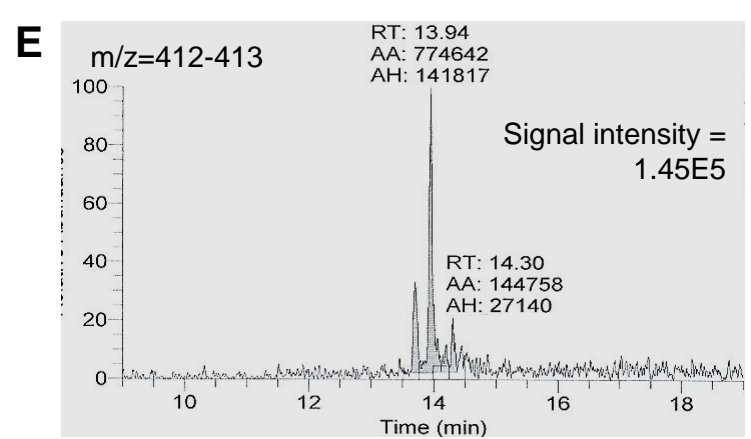

Supplement: Figure S15 — Analysis of sterols in c14dm − amastigotes. Total lipids from c14dm − amastigotes were analyzed by GC-MS. (A) TIC chromatogram of lipids with m/z of 50–500. Std: internal standard (cholesta-3,5-diene). (B) Selected ion monitoring of sterol species with m/z of 386–387 (cholesterol). (C) Selected ion monitoring of sterol species with m/z of 396–397 (ergosterol and 5-dehydroepisterol). (D) Selected ion monitoring of sterol species with m/z of 398–399 (mainly14-methyl-zymosterol). (E) Selected ion monitoring of sterol species with m/z of 412–413 (14-methyl-fecosterol). Retention times for major peaks are marked in A–E. Signal intensity is indicated in each panel. (PDF) [file ppat.1004427.s015.pdf]

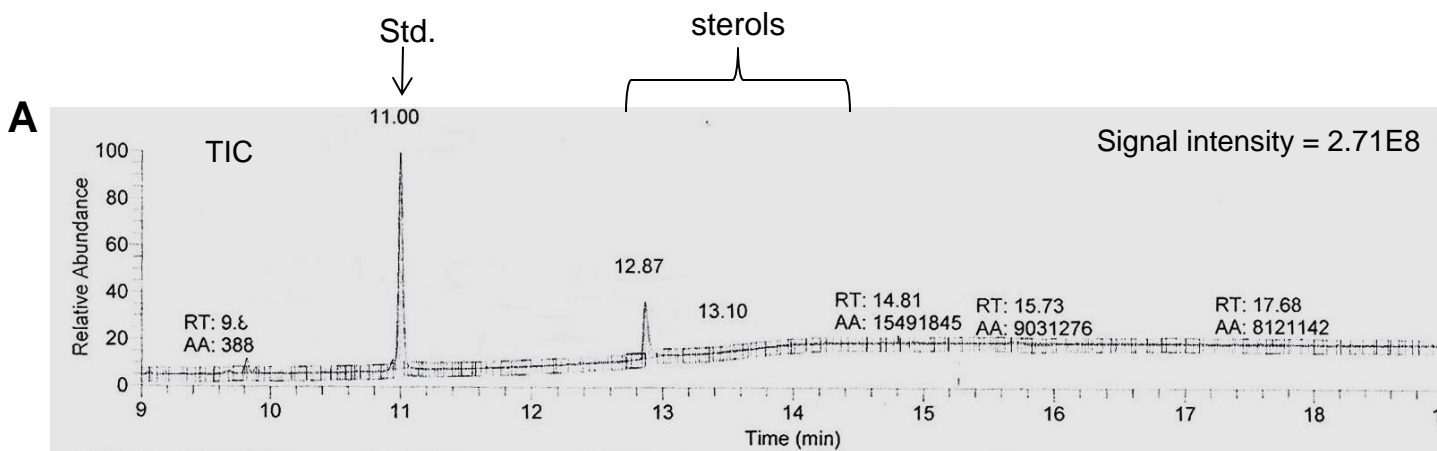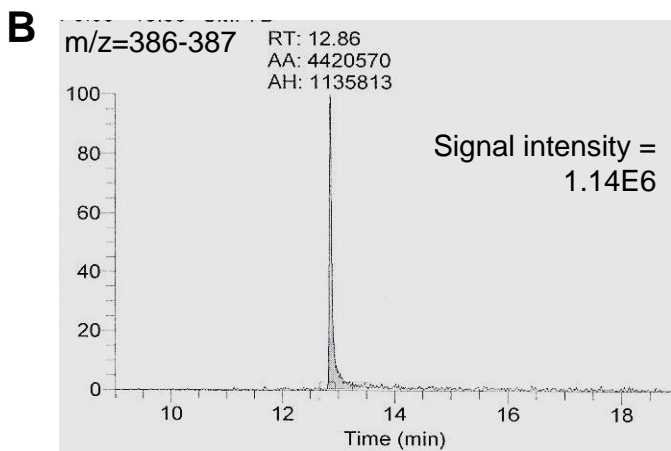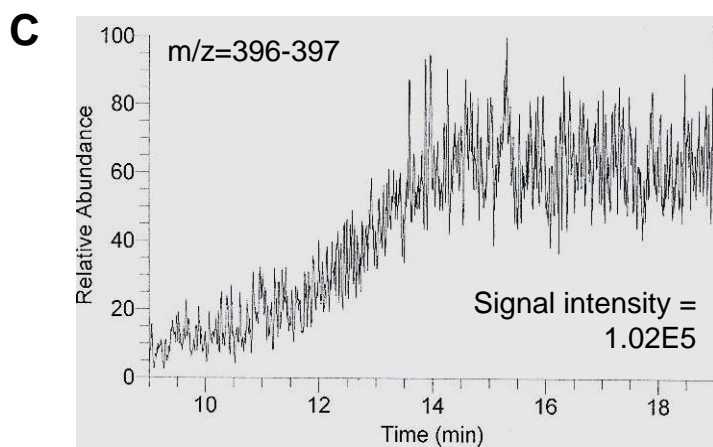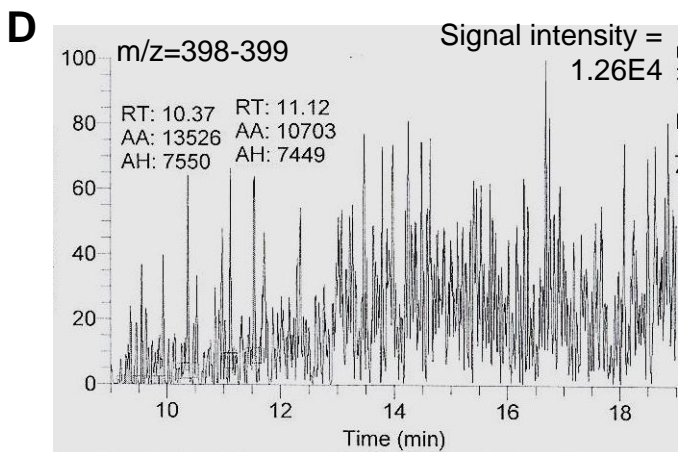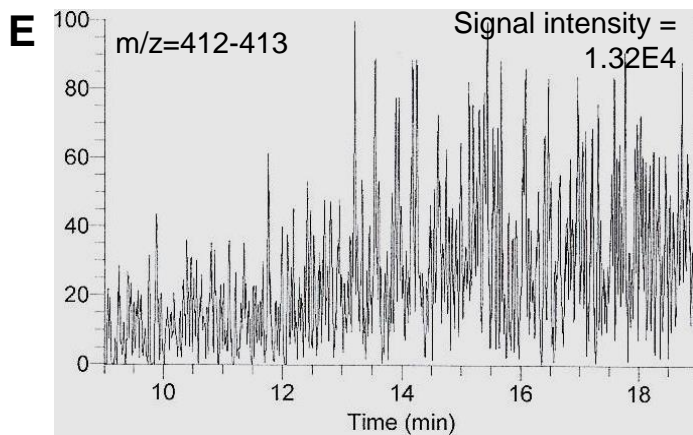

Supplement: Figure S16 — Analysis of sterols in uninfected mouse tissue. Total lipids from uninfected mouse footpads were analyzed by GC-MS. (A) TIC chromatogram of lipids with m/z of 50–500. Std: internal standard (cholesta-3,5-diene). (B) Selected ion monitoring of sterol species with m/z of 386–387 (cholesterol). (C) Selected ion monitoring of sterol species with m/z of 396–397 (ergosterol and 5-dehydroepisterol). (D) Selected ion monitoring of sterol species with m/z of 398–399 (episterol). (E) Selected ion monitoring of sterol species with m/z of 412–413 (14-methyl-fecosterol). Retention times for major peaks are marked in A–E. Signal intensity is indicated in each panel. (PDF) [file ppat.1004427.s016.pdf]

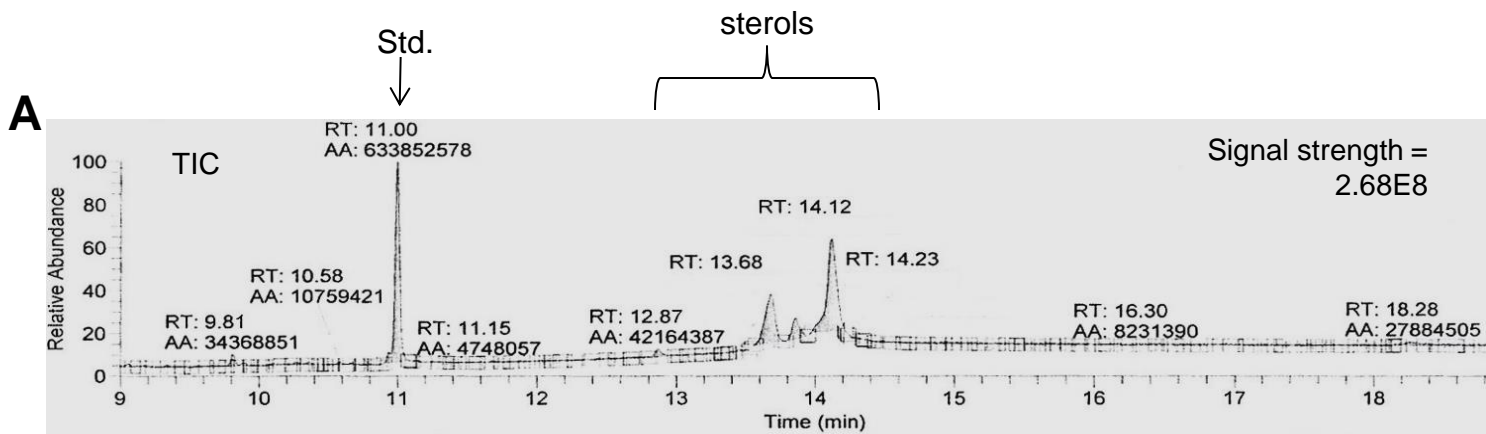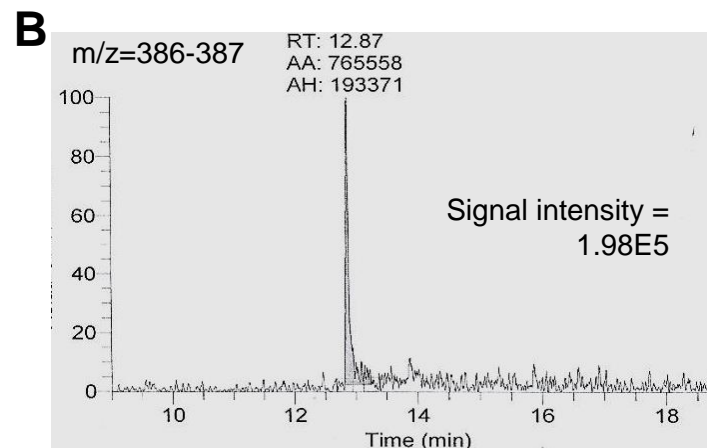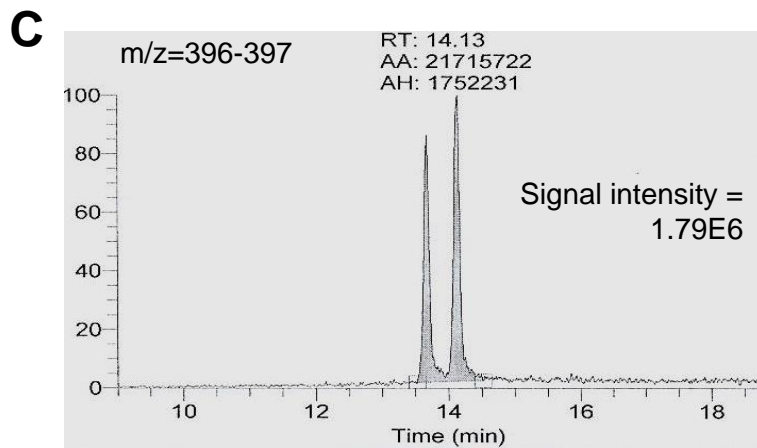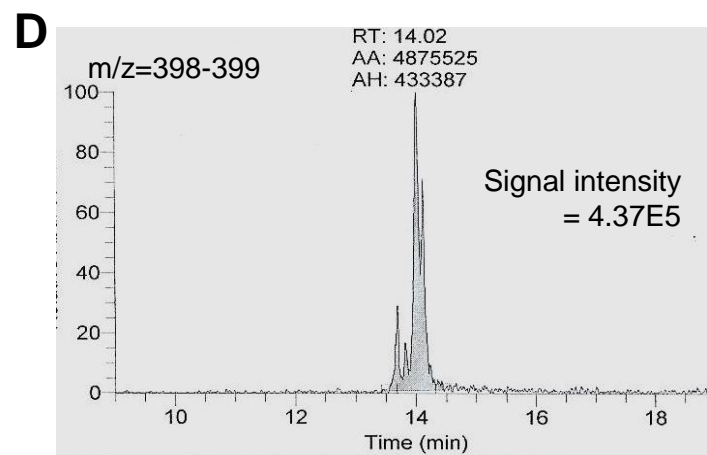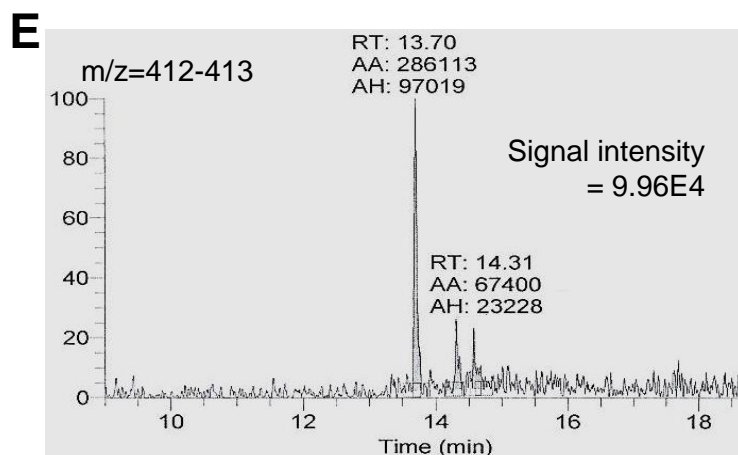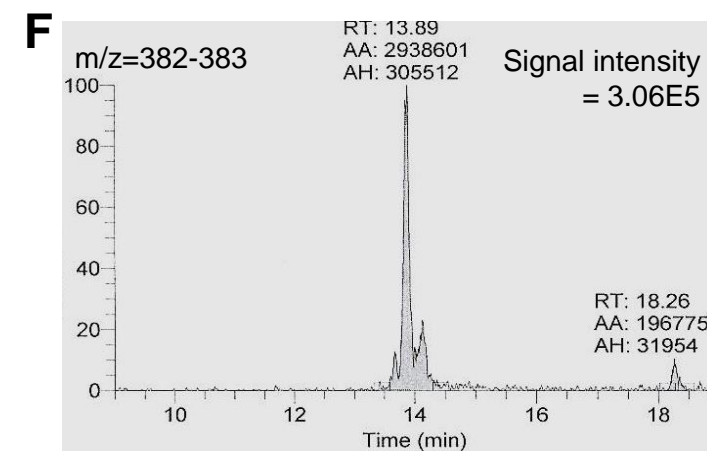

Supplement: Figure S17 — Analysis of sterols in WT promastigotes. Total lipids from WT promastigotes were analyzed by GC-MS. (A) TIC chromatogram of lipids with m/z of 50–500. Std: internal standard (cholesta-3,5-diene). (B)–(F) Selected ion monitoring of sterol species with m/z of 386–387 (B), 396–397 (C), 398–399 (D), 412–413 (E), and 382–383 (F). Retention times for major peaks are marked in A–E. Signal intensity is indicated in each panel. (PDF) [file ppat.1004427.s017.pdf]

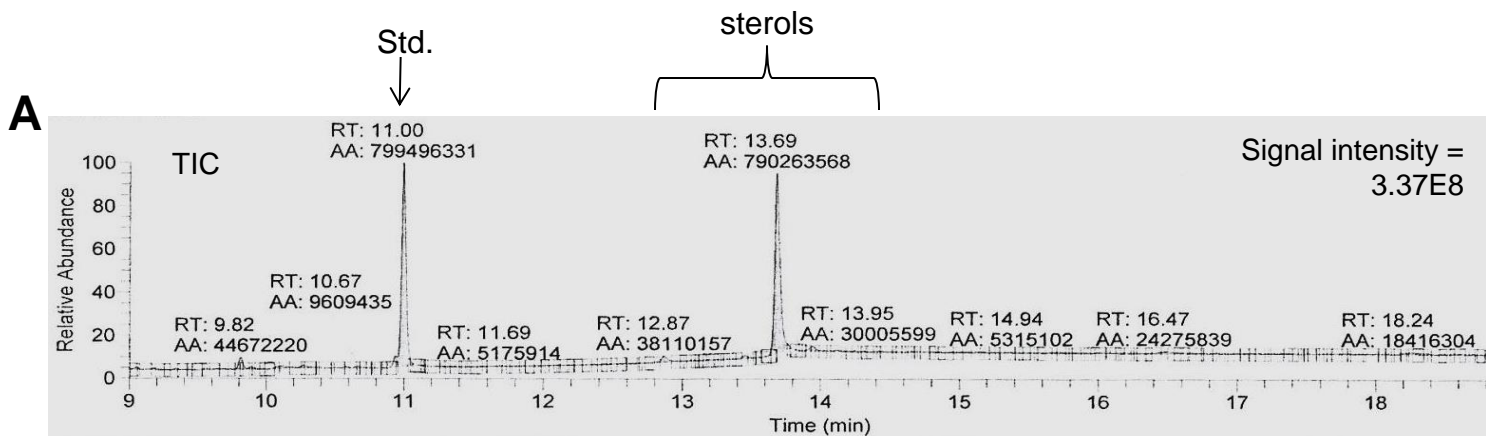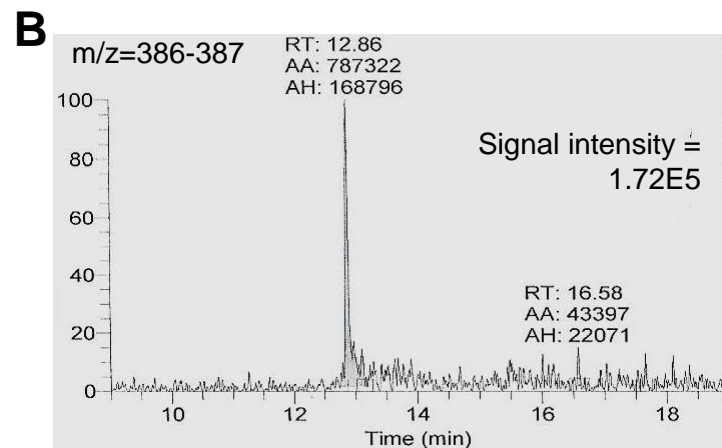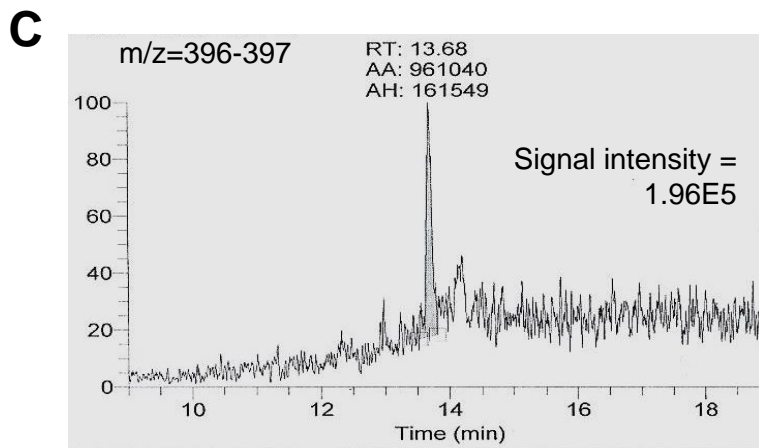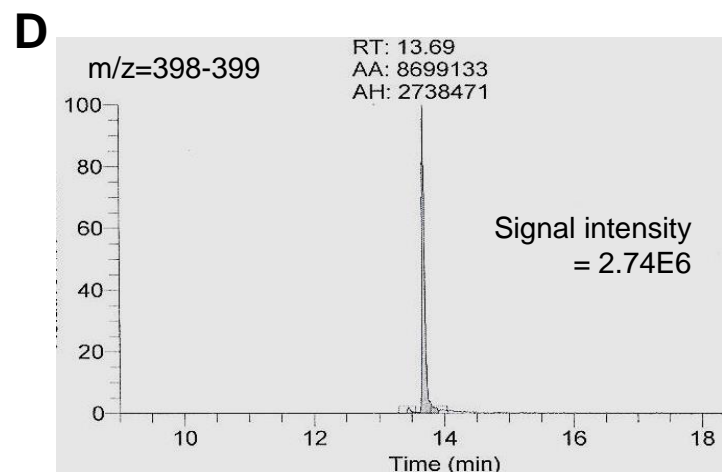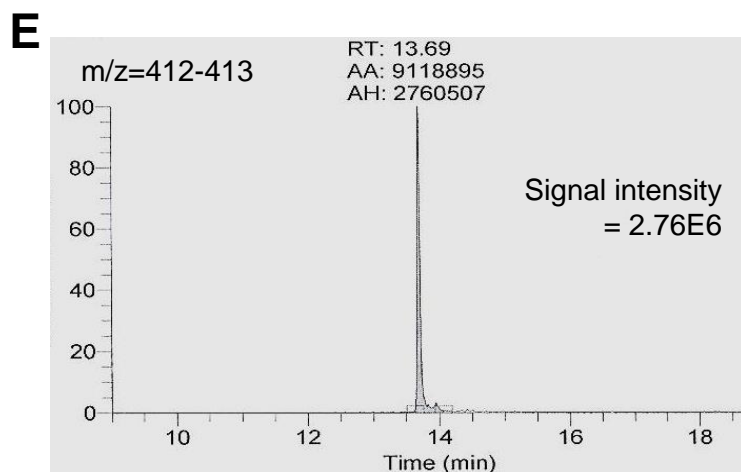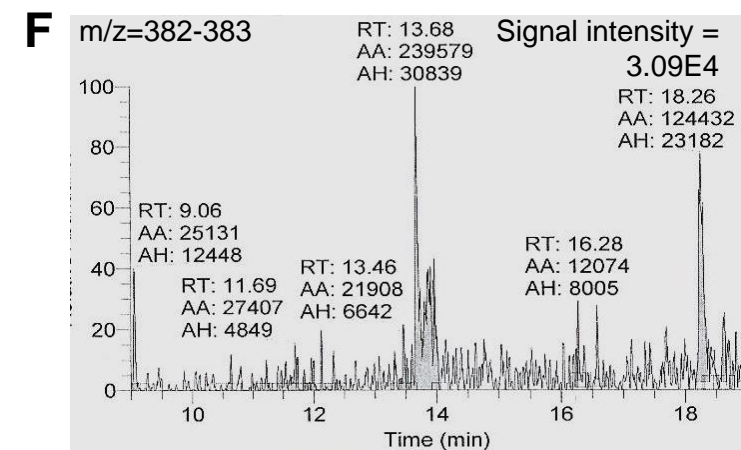

Supplement: Figure S18 — Analysis of sterols in c14dm − promastigotes. Total lipids from c14dm − promastigotes were analyzed by GC-MS. (A) TIC chromatogram of lipids with m/z of 50–500. Std: internal standard (cholesta-3,5-diene). (B)–(F) Selected ion monitoring of sterol species with m/z of 386–387 (B), 396–397 (C), 398–399 (D), 412–413 (E), and 382–383 (F). Retention times for major peaks are marked in A–E. Signal intensity is indicated in each panel. (PDF) [file ppat.1004427.s018.pdf]

**A**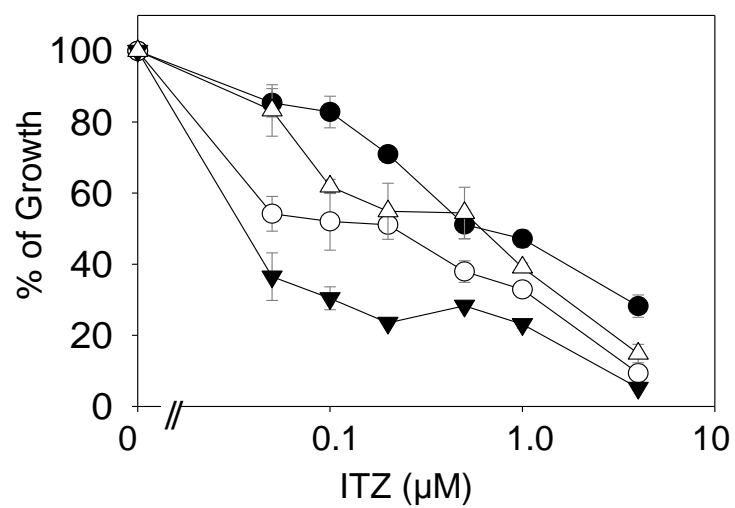**B**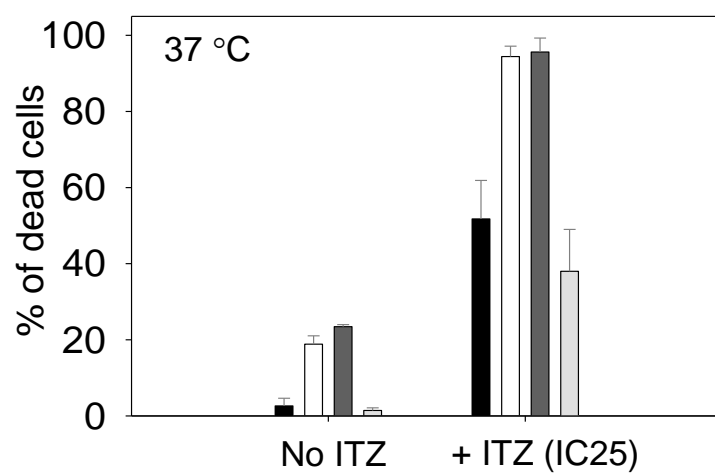

Supplement: Figure S19 — ITZ treatment leads to extreme sensitivity to heat in several Leishmania species. (A) Log phase promastigotes of L. major LV39 (black circle), L. amazonensis (white circle), L. mexicana (black triangle) and L. donovani (white triangle) were cultured in various concentrations of ITZ. Culture densities were determined after 48 hours. (B) Promastigotes were grown in the absence or presence of ITZ (at IC25 concentrations) to stationary phase. Cells were then incubated at 37°C/5% CO2 and cell viability was measured after 8 hours (black bars: L. major LV39, white bars: L. amazonensis, dark grey bars: L. mexicana, light grey bar: L. donovani). Experiments were repeated three times and error bars represent standard deviations. (PDF) [file ppat.1004427.s019.pdf]

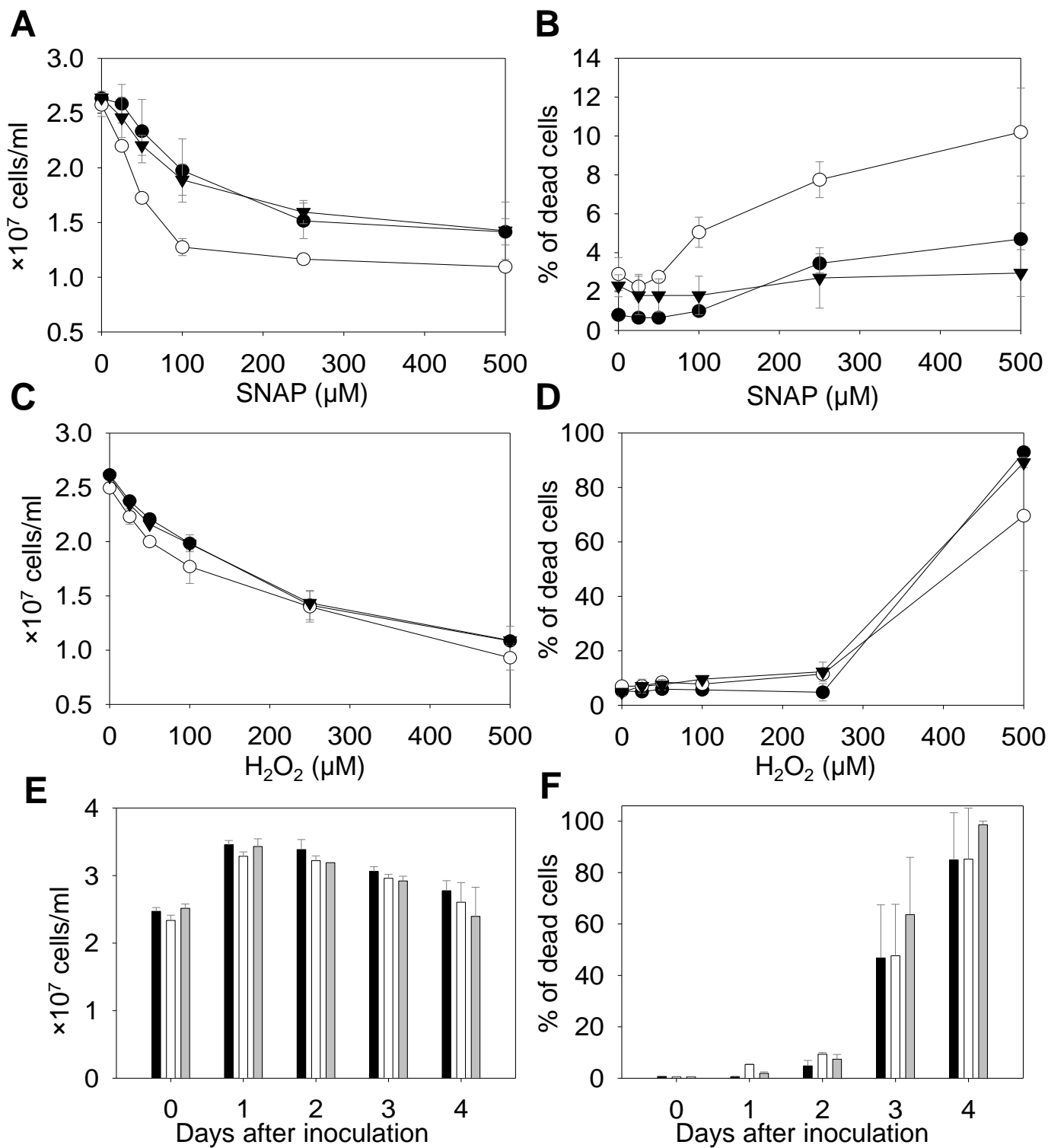

Supplement: Figure S20 — Ability of c14dm − mutants to survive nitrosative, oxidative and acidic pH stress. Stationary phase promastigotes of WT (black circle, black bars), c14dm − (white circle, white bars), and c14dm − /+c14dm (black triangle, grey bars) were treated with various concentrations of SNAP (A–B), H2O2 (C–D), or incubated in pH 5.0 media (E–F). Cell density and percentage of dead cells were measured after 48 hours. Experiments were repeated three times and error bars represent standard deviations. (PDF) [file ppat.1004427.s020.pdf]
