# Supplementary material for: Sterol Biosynthesis Is Required for Heat Resistance but Not Extracellular Survival in Leishmania
Source: PLoS Pathog. 2014 Oct 23;10(10):e1004427. doi: 10.1371/journal.ppat.1004427 (PMC4207814; doi:10.1371/journal.ppat.1004427)
Supplement: Figure S2 — Alignment of C14DMs from L. major (LmjF11.1100), H. sapiens (Q16850), A. fumigatus (XP_752137), C. albicans (XP_716822), and M. tuberculosis (NP_215278). Highly conserved residues are highlighted in black. The asterisks mark the predicted sterol substrate binding site and the box indicates the predicted heme binding motif. (PDF) [file ppat.1004427.s002.pdf]

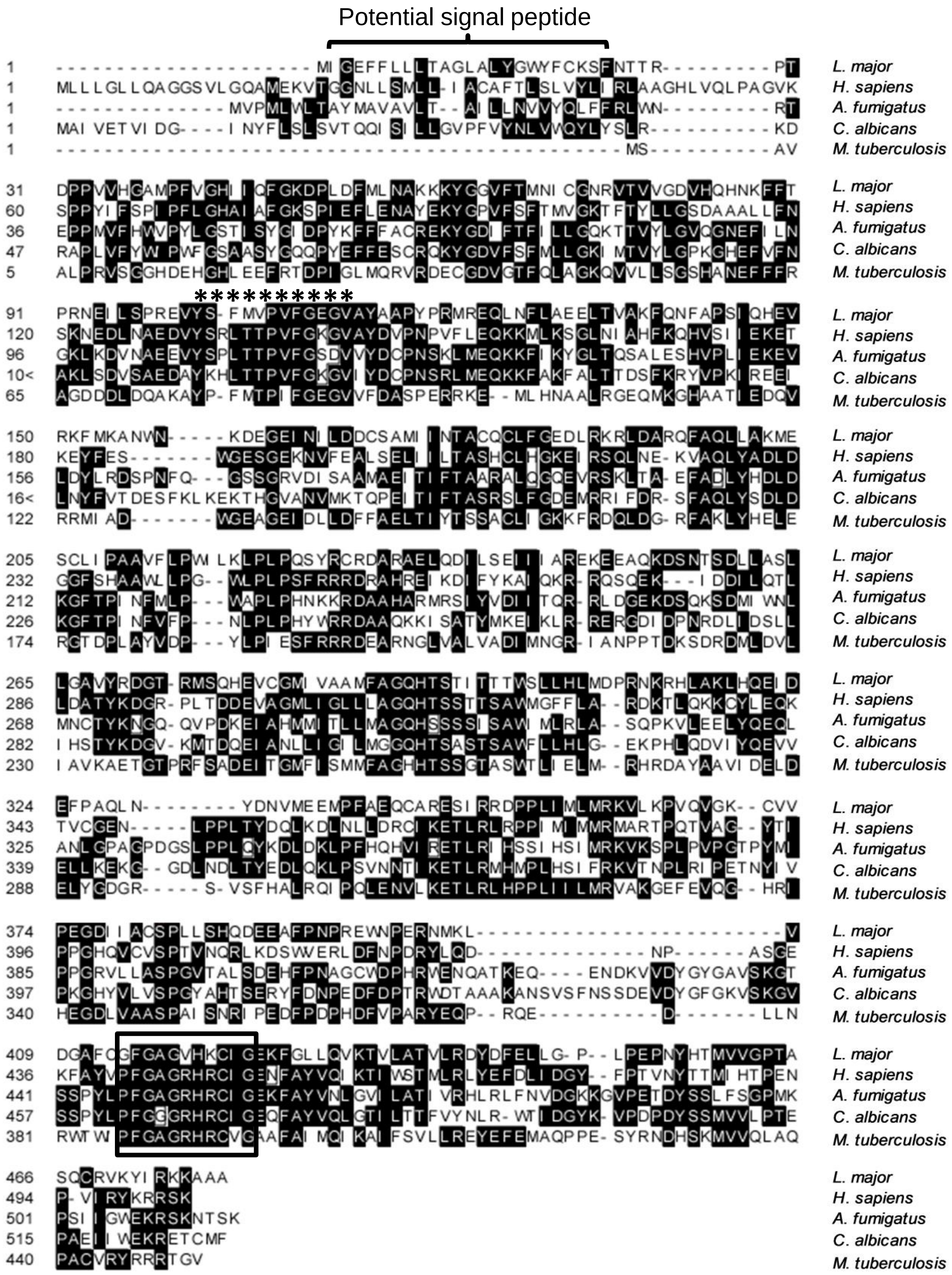

Potential signal peptide
L. major
H. sapiens
A. fumigatus
C. albicans
M. tuberculosis
