# Supplementary material for: Sterol Biosynthesis Is Required for Heat Resistance but Not Extracellular Survival in Leishmania
Source: PLoS Pathog. 2014 Oct 23;10(10):e1004427. doi: 10.1371/journal.ppat.1004427 (PMC4207814; doi:10.1371/journal.ppat.1004427)

14-Methyl fecosterol (412.6) +14-Methyl zymosterol (398.6)

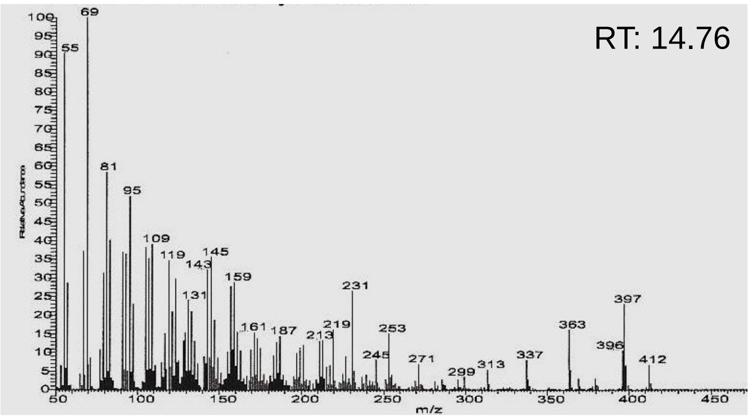

Ergosterol (396.6)

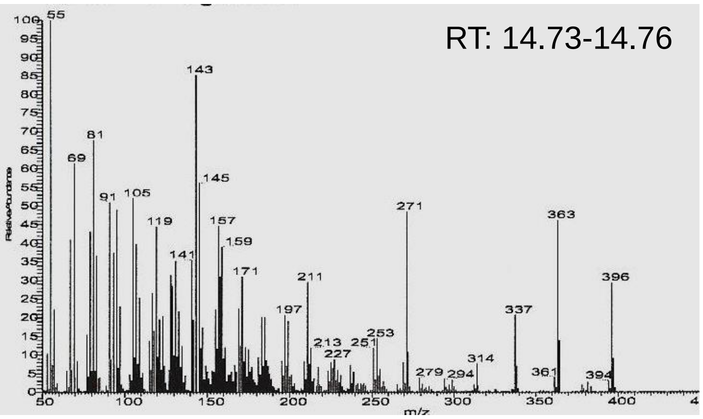

Cholesta-3,5-diene (368.7)

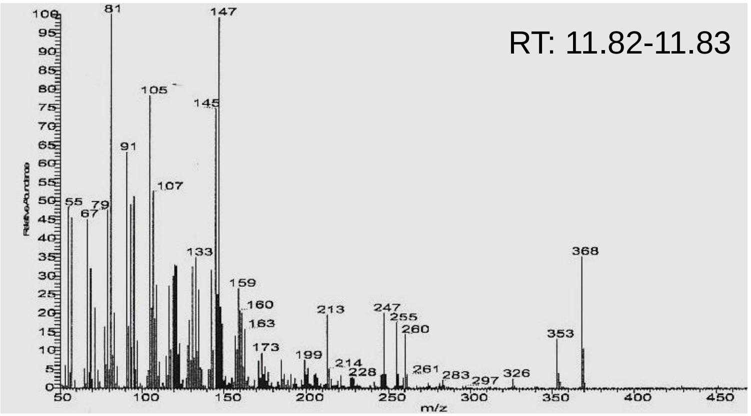

Episterol (398.6)

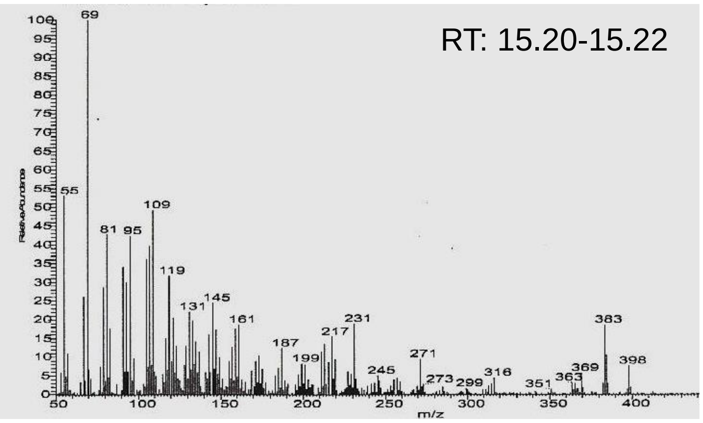

5-Dehydroepisterol (396.6)

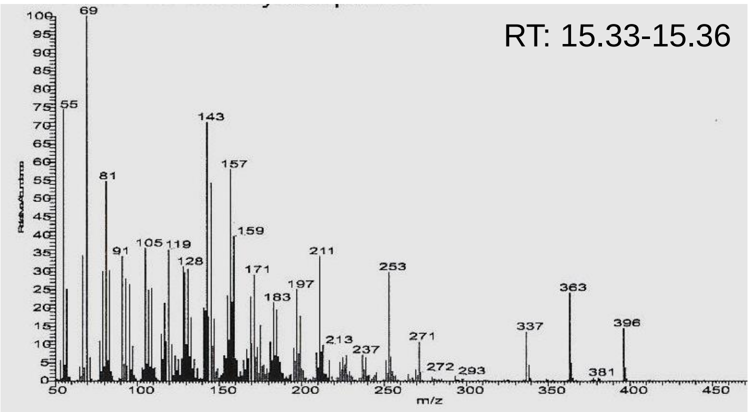

Cholesterol (386.6)

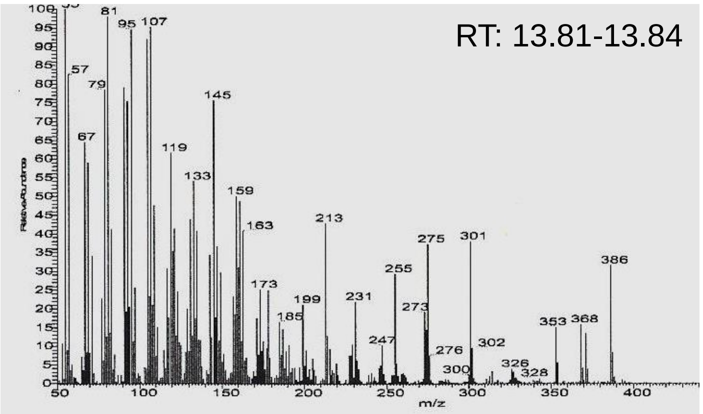

Cholesta-5,7,24-trienol (382.6)

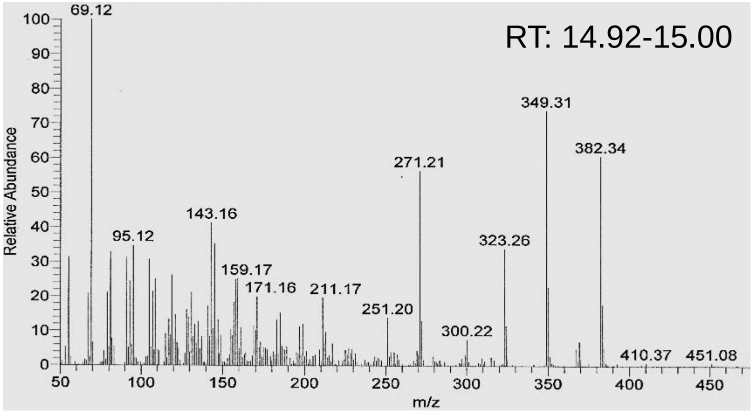

Supplement: Figure S9 — Electron ionization mass spectra of Leishmania sterols. Lipids from WT or c14dm − promastigotes were analyzed by GC-MS. Electron ionization spectra (70 eV) of sterol species based on retention time (RT) in Fig. S5–S8 were included. The predicted sterol types were indicated above each panel. (PDF) [file ppat.1004427.s009.pdf]
