# Supplementary material for: Sterol Biosynthesis Is Required for Heat Resistance but Not Extracellular Survival in Leishmania
Source: PLoS Pathog. 2014 Oct 23;10(10):e1004427. doi: 10.1371/journal.ppat.1004427 (PMC4207814; doi:10.1371/journal.ppat.1004427)
Supplement: Table S1 — Sterol composition in Leishmania promastigotes. Promastigotes were cultured in the absence or presence of ITZ (provided at IC25 concentrations: 120 nM for L. major, 81 nM for L. donovani, 3.3 nM for L. mexicana, and 25 nM for L. amazonensis) from early log phase to day 1 stationary phase and total lipids were analyzed by GC-MS. Abundances of ergosterol, 5-dehydroepisterol, cholesterol, cholesta-5,7,24-trienol, 14-methyl sterols (14-methylfecosterol + 14-methylzymosterol), and total sterols were estimated in relation to the internal standard cholesta-3,5-diene (provided at 2.0×10^7^ molecules/cell). Analyses were repeated 3 times and averaged values ± standard deviations (SDs) were shown. ND: not detectable. (PDF) [file ppat.1004427.s021.pdf]

Table S1. Sterol composition in *Leishmania* promastigotes

	Ergosterol	5-Dehydro-episterol	Cholesterol	Cholesta-5,7,24-trienol	14-Methyl sterols	Total
L. major	1.1±0.2	2.2±0.3	0.17±0.02	0.43±0.02	ND	3.9±0.5
L. major +ITZ	ND	ND	0.19±0.07	ND	4.0±0.4	4.2±0.5
c14dm¯	ND	ND	0.14±0.04	ND	3.8±0.3	3.9±0.4
c14dm¯/+C14DM	1.0±0.3	2.1±0.2	0.20±0.01	0.24±0.04	ND	3.6±0.5
L. donovani	0.9±0.3	2.0±0.5	0.21±0.04	1.3±0.13	ND	4.4±0.9
L. donovani +ITZ	ND	ND	0.24±0.06	ND	4.0±0.3	4.3±0.4
L. mexicana	0.26±0.11	4.0±0.2	0.20±0.04	0.29±0.17	ND	4.7±0.6
L. mexicana +ITZ	ND	ND	0.15±0.01	ND	3.0±0.6	3.2±0.6
L. amazonensis	0.32±0.1	3.8±0.2	0.17±0.02	1.1±0.5	ND	5.4±0.8
L. amazonensis +ITZ	ND	ND	0.18±0.03	ND	4.1±0.2	4.3±0.3

Promastigotes were cultured in the absence or presence of ITZ (provided at IC25 concentrations: 120 nM for *L. major*, 81 nM for *L. donovani*, 3.3 nM for *L. mexicana*, and 25 nM for *L. amazonensis*) from early log phase to day 1 stationary phase and total lipids were analyzed by GC-MS. Abundances of ergosterol, 5-dehydroepisterol, cholesterol, cholesta-5,7,24-trienol, 14-methyl sterols (14-methylfecosterol + 14-methylzymosterol), and total sterols were estimated in relation to the internal standard cholesta-3,5-diene (provided at 2.0×10^7 molecules/cell). Analyses were repeated 3 times and averaged values $\pm$ standard deviations (SDs) were shown. ND: not detectable.
