# Supplementary material for: Sterol Biosynthesis Is Required for Heat Resistance but Not Extracellular Survival in Leishmania
Source: PLoS Pathog. 2014 Oct 23;10(10):e1004427. doi: 10.1371/journal.ppat.1004427 (PMC4207814; doi:10.1371/journal.ppat.1004427)
Supplement: Table S2 — Susceptibility of Leishmania parasites to ITZ (IC values in µM). Promastigotes were inoculated at 2.0×10^5^ cells/ml in various concentrations of ITZ and culture densities were determined after 48 hours. IC25, IC50, and IC90 are ITZ concentrations that inhibit growth by 25%, 50%, and 90%, respectively, in comparison to control cultures (no ITZ). Experiments were performed three times (average ± SD). (PDF) [file ppat.1004427.s022.pdf]

Table S2. Susceptibility of *Leishmania* parasites to ITZ (IC values in μM)

	IC25	IC50	IC90
L. major	0.12±0.10	0.40±0.10	10.0±1.4
L. amazonensis	0.025±0.006	0.1±0.1	4.0±0.8
L. mexicana	0.0033±0.002	0.01±0.005	1.2±0.2
L. donovani	0.081±0.010	0.5±0.2	5.0±1.7

Promastigotes were inoculated at 2.0 x 10^5 cells/ml in various concentrations of ITZ and culture densities were determined after 48 hours. IC25, IC50, and IC90 are ITZ concentrations that inhibit growth by 25%, 50%, and 90%, respectively, in comparison to control cultures (no ITZ). Experiments were performed three times (average±SD).
