# Supplementary material for: Sterol Biosynthesis Is Required for Heat Resistance but Not Extracellular Survival in Leishmania
Source: PLoS Pathog. 2014 Oct 23;10(10):e1004427. doi: 10.1371/journal.ppat.1004427 (PMC4207814; doi:10.1371/journal.ppat.1004427)
Supplement: Table S3 — List of oligonucleotides used in this study. Sequences in lowercase represent restriction enzyme recognition sites. (PDF) [file ppat.1004427.s023.pdf]

Table S3. List of oligonucleotides used in this study.

Primer #	Name	Sequence
170	5' ORF C-14 Demethylase	GATCATagatctACCATGATCGGCGAGTTCTTCCT
171	3' ORF C-14 Demethylase	GATCATagatctCTAAGCAGCCGCCTTCTTCC
172	5' 5' UTR C-14 Demethylase	GATCATgaattcCGCCCGCGGATTTCGTGAAG
173	3' 5' UTR C-14 Demethylase	GTCAGCggatccGATCTAactagtGATGGCGAACCCTTCTCGGC
174	5' 3' UTR C-14 Demethylase	GATCATggatccAGCGAGGACGCGGGCTGG
175	3' 3' UTR C-14 Demethylase	GATCATaagcttGTGCAGCAACAGTCAGTTTT
176	5' Southern probe C-14 DM	TAGTGCGACGGTGATTGCCG
177	3' Southern probe C-14 DM	GCTTGCCTGGGCGCTCGGGC
178	C-14 Demethylase Internal I	CCGCGCCATACCCGCGCATG
179	C-14 Demethylase Internal II	CGGCGGGTCACGGCGGATCG
333	c14dm ORF reverse+GFP	GATCATagatctAGCAGCCGCCTTCTTCCTGA
7	5' ORF pXG Upstream	CACTTTCAAGGCTTCCCGAAC
8	3' ORF pXG Downstream	CGCGAATTGAAGAACAGAGAAGC
336	seqing pxg-c14dm-GFP FOR	CACCATCTACGCGTGCACATCATC
337	seqing pxg-c14dm-GFP REV	ACGCCGTAGGTGAAGGTGGTC

Sequences in lowercase represent restriction enzyme recognition sites.
